# Nox4 and Duox1/2 Mediate Redox Activation of Mesenchymal Cell Migration by PDGF

**DOI:** 10.1371/journal.pone.0154157

**Published:** 2016-04-25

**Authors:** Pyotr A. Tyurin-Kuzmin, Nadezhda D. Zhdanovskaya, Anna A. Sukhova, George D. Sagaradze, Eugene A. Albert, Ludmila V. Ageeva, George V. Sharonov, Alexander V. Vorotnikov, Vsevolod A. Tkachuk

**Affiliations:** 1 Department of Biochemistry and Molecular Medicine, Faculty of Fundamental Medicine, M.V. Lomonosov Moscow State University, Moscow, Russia; 2 Institute of Experimental Cardiology, Russian Cardiology Research Center, Moscow, Russia; 3 Laboratory of Optical Microscopy and Spectroscopy of Biomolecules, Department of Structural Biology, Shemyakin-Ovchinnikov Institute of Bioorganic Chemistry, Moscow, Russia; University of Sassari, ITALY

## Abstract

Platelet derived growth factor (PDGF) orchestrates wound healing and tissue regeneration by regulating recruitment of the precursor mesenchymal stromal cells (MSC) and fibroblasts. PDGF stimulates generation of hydrogen peroxide that is required for cell migration, but the sources and intracellular targets of H_2_O_2_ remain obscure. Here we demonstrate sustained live responses of H_2_O_2_ to PDGF and identify PKB/Akt, but not Erk1/2, as the target for redox regulation in cultured 3T3 fibroblasts and MSC. Apocynin, cell-permeable catalase and LY294002 inhibited PDGF-induced migration and mitotic activity of these cells indicating involvement of PI3-kinase pathway and H_2_O_2_. Real-time PCR revealed Nox4 and Duox1/2 as the potential sources of H_2_O_2_. Silencing of Duox1/2 in fibroblasts or Nox4 in MSC reduced PDGF-stimulated intracellular H_2_O_2_, PKB/Akt phosphorylation and migration, but had no such effect on Erk1/2. In contrast to PDGF, EGF failed to increase cytoplasmic H_2_O_2_, phosphorylation of PKB/Akt and migration of fibroblasts and MSC, confirming the critical impact of redox signaling. We conclude that PDGF-induced migration of mesenchymal cells requires Nox4 and Duox1/2 enzymes, which mediate redox-sensitive activation of PI3-kinase pathway and PKB/Akt.

## Introduction

Increased migration and proliferation of mesenchymal cells critically contributes to wound healing, tissue repair and maintenance of homeostasis [1]. The inflammatory step involves oxidative stress and reactive oxygen species (ROS) [1]; it is followed by the reparatory steps mediated by mesenchymal cells such as MSC and fibroblasts [2,3]. Platelet derived growth factor (PDGF) is the major chemoattractant that guides these mesenchymal cells into injured areas where it stimulates their proliferation and extracellular matrix production [4,5].

In mesenchymal cells, PDGF acts through PI3-kinase (PI3K) and p44 and p42 extracellular signal-regulated kinase (Erk1/2) pathways [5], which both are involved in migration [6,7] and mitotic responses [8]. The PI3K pathway controls polarized cell morphology critical to migration [9,10] and mediates cell cycle progression into the S-phase [8]. It targets activation of protein kinase B (PKB) commonly known as Akt, by increased phosphorylation of two critical sites that report activation of PKB/Akt [10,11]. The Erk1/2 pathway mainly controls mitotic activity, survival and differentiation [12]. It may be also involved in migration via the effects on cytoskeletal and focal adhesion dynamics [13].

PDGF receptors also trigger generation of ROS and H_2_O_2_ [14,15], which upregulate the downstream signaling from the receptors by inhibiting tyrosine phosphatases [16] and activating tyrosine kinases [17]. Thus, H_2_O_2_ was found required for PDGF-induced migration of fibroblasts [18]. However whether migration of MSC is redox dependent and what are the intracellular targets for H_2_O_2_ is unknown.

NADPH-oxidases (Nox) and dual oxidases (Duox) are the major receptor-dependent sources of H_2_O_2_ in cells [14,15,19]. Currently there is no consensus as to which Nox/Duox enzymes are involved in cell migration and whether this is cell type specific. The vascular smooth muscle cells and endothelial cells involve Nox1 and Nox4 in migration [20,21], whereas leukocytes seem to use Duox1/2 [22,23]. These studies are largely restricted by unavailability of specific Nox inhibitors and specific probes reporting live dynamics of H_2_O_2_ [14]. As a result, few compounds are widely used as general, broad specificity Nox inhibitors or ROS scavengers, such as diphenyleneiodonium (DPI) and ebselen. In addition, apocynin (4-hydroxy-3-methoxyacetophenone) is widely used as the Nox assembly inhibitor and has been reported as an antioxidant [24,25]. Thus, little is known whether and which NADPH-oxidases control migration of mesenchymal cells such as MSC and fibroblasts.

In this paper we show that PDGF activates migration and mitotic activity of NIH-3T3 fibroblasts and human primary MSC via accumulation of H_2_O_2_ and redox-dependent activation of PI3K pathway. In contrast, PDGF activated the Erk1/2 pathway in redox-independent manner and the Erk1/2 activation was dispensible for migration of fibroblasts. Apocynin blocked PDGF-induced accumulation of intracellular H_2_O_2_, phosphorylation of PKB/Akt, migration and mitotic activity, but had no effect on the Erk1/2 activation. Silencing of Duox1/2 expression in fibroblasts and that of Nox4 in MSC reduced cytoplasmic H_2_O_2_, PDGF-stimulated phosphorylation of PKB/Akt and migration. The critical contribution of redox mechanisms to mesenchymal cell migration was confirmed in a comparative mode. In contrast to PDGF, epidermal growth factor (EGF) failed to stimulate intracellular H_2_O_2_, phosphorylation of PKB/Akt, and migration of 3T3 fibroblasts. Still, EGF effectively activated the Erk1/2 pathway and mitotic activity in redox-independent fashion. These results show that sustained accumulation of cytoplasmic H_2_O_2_ in mesenchymal cells is a part of specific response to PDGF; it provides for redox regulation of migration and mitotic activity via PI3K pathway, whereas the Erk1/2 pathway only controls mitotic activity in redox insensitive manner.

## Materials and Methods

### Reagents and antibodies

Apocynin (acetovanillone), PEG-catalase and ebselen were purchased from Sigma (USA). PDGF-BB and EGF were from Cell Signaling (USA). DPI, U0126, LY294002 were from Calbiochem (Germany). The primary antibodies to total Akt, phospho-Akt (Ser-473), phospho-Erk1/2 (Thr202/Tyr204), total Erk1/2, and secondary antibody were from Cell Signaling (USA). Antibody to Nox4, Duox1 and Duox 2 were from Novus Biologicals (USA), antibody to vinculin were from Sigma (USA). The expression plasmid encoding H_2_O_2_ biosensor HyPer fused to the nuclear export sequence (HyPer-NES) was from Evrogen, Russia. DCF-DA was kindly donated by Dr. M.Yu. Menshikov (Cardiology Research Center, Moscow, Russia).

### Cell culture and transfection

NIH-3T3 fibroblasts (obtained from EMBL, Germany, and Russian Cell Culture Collection, RAS Institute of Cytology, St-Petersburg, Russia) were cultured at 37°C and 5% CO_2_ in DMEM (Dulbecco’s Modified Eagle Medium) containing low glucose (1 g/l) and supplemented with 10% fetal bovine serum (FBS), 100 U/ml penicillin and 100 μg/ml streptomycin. Before transfection the cells were plated on Nunc Lab-Tek coverglasses (Thermo Scientific, USA) for 12–24 h. Transfection was performed using the FuGene 6 Transfection Reagent (Roche Molecular Biochemicals, Germany) according to the manufacturer’s protocol. One hour prior to transfection the cell medium was changed to DMEM supplemented with 0.5% FBS and no antibiotics. The medium was changed back to the fully supplemented DMEM 4–14 h after transfection. The cells were assayed 36–48 h after transfection. HEK 293T cells were cultured in the same manner and plated onto 100 mm plates at 3*10^6^ cells per plate 24 hours prior to calcium phosphate transfection. Human primary adipose derived mesenchymal stromal cells (MSC) were kindly donated by Olga Grigorieva (Dept of Biochemistry and Molecular Medicine, Lomonosov Moscow State University, Moscow, Russia). MSC were cultured in AdvanceStem basal medium (ASBM) for undifferentiated human mesenchymal stem cells (GE Healthcare, USA) containing 10% Advance Stem Cell Growth Supplement (GE Healthcare, USA). The MSC were isolated from 8 donors and used in independent experiments.

### shRNA plasmid preparation

To construct shRNA expressing plasmids the following sequences for oligonucleotides were acquired from TRC database (Broad institute): Nox4, forward, *CCGGGCATCA AATAACCACC TGTATCTCGA GATACAGGTG GTTATTTGAT GCTTTTTG*, Nox4 reverse, *AATTCAAAAA GCATCAAATA ACCACCTGTA TCTCGAGATA CAGGTGGTTA TTTGATGC*, Duox1, forward, *CCGGGCCTTA ACCCAGTACT CATTTCTCGA GAAATGAGTA CTGGGTTAAG GCTTTTTG*, Duox1, reverse, *AATTCAAAAA GCCTTAACCC AGTACTCATT TCTCGAGAAA TGAGTACTGG GTTAAGGC*, scramble shRNA, forward, *CCGGCCTAAG GTTAAGTCGC CCTCGCTCGA GCGAGGGCGA CTTAACCTTA GGTTTTTG*, scramble shRNA, reverse, *AATTCAAAAA CCTAAGGTTA AGTCGCCCTC GCTCGAGCGA GGGCGACTTA ACCTTAGG*. The sequences were synthesized by Evrogen (Evrogen, Russia) as single strand DNA oligonucleotides and annealed afterwards. The pLKO.1-mCherry vector was constructed from the lentivirus plasmid pLKO.1 (Addgene plasmid 10878, Addgene, USA) by replacing the sequence encoding puromycin resistance with the sequence encoding mCherry reporter protein. The above oligonucleotides were inserted into the pLKO.1-mCherry vector using EcoRI and AgeI restriction enzymes.

### Generation of Nox4 and Duox1/2 deficient cell lines

To generate the shRNA producing lentiviral particles two 60 mm plates with subconfluent HEK 293 cells were cotransfected with the pLKO.1-mCherry plasmid bearing either the shNox4, or shDuox1, or scramble insert described above, the envelop vector and the packaging vector (Addgene, USA). After 12 hours the medium containing lentiviral particles was collected and exchanged for the fully supplemented DMEM. It was similarly collected and replaced twice more at 24 and 48 hours post transfection. The supernatants were combined, filtered through the 0.45 μm sterile filter and used to transduce NIH-3T3 fibroblasts that were plated on 35 mm Petri dishes 24 hours beforehand. The fibroblast culture medium was replaced by 1.5 ml of the combined supernatants supplemented with 8 μg/ml (final) of polybrene. After 12 h the medium was exchanged for another 1.5 ml portion of the lentiviral supernatant with polybrene and cells were cultured for 24 h more. Then medium was exchanged for the fully supplemented DMEM, the cells were propagated for another 3 days and examined for mCherry fluorescence. mCherry positive cells were selected by fluorescence activated cell sorting on FACSAria III sorter (BD Bioscienecs, USA) with 30 psi pressure and 85 μm nozzle. The 561 nm excitation laser and 610/20 emission filter were used.

### siRNA-mediated silencing of Nox/Duox expression

NIH-3T3 fibroblasts and MSC were seeded in 12 well plates 1 day before the transfection at the 60–80% of confluence. The siRNA oligos were obtained from Dharmacon (GE Life Sciences, USA) and Syntol (Russia). NIH-3T3 cells were transfected with 25 nM siRNA using Dharmafect reagent (GE Life Sciences, USA) according to the manufacturer's protocol. Human MSC were transfected with 5 nmol siRNA using Lipofectamine RNAiMAX (Thermo Fisher Scientific Inc., USA) according to the manufacturer's protocol. Measurements of cell migration, intracellular H_2_O_2_, and phosphorylation of PKB/Akt and Erk1/2 were conducted at 72–96 hours post-transfection. The expression levels of Nox/Duox targets of siRNAs were determined by western blots in the same lysates. The targeted protein mRNAs were additionally determined by real-time polymerase chain reaction (RT-PCR) 48 h post transfection.

### RNA Isolation and Quantitative RT-PCR

Total RNA was isolated using TRIzol® Reagent (Life Technologies, USA) and purified using the Direct-zol™ RNA MiniPrep (Zymo Research, USA) according to the manufacturer’s instructions. On-column DNase digestion of samples was performed. The RNA was quantified and qualified using Nanodrop spectrophotometer (Thermo Scientific, USA). The total RNA samples with the 260/280 nm ratio of 1,9 to 2,1 were used for further analyses. The first strand cDNA was synthesized from 1 μg of total RNA with the SuperScript ® III First-Strand Synthesis System (Life Technologies, USA) in a 20 μl reaction using Oligo(dT)_20_ primers according to the manufacturer’s instructions. The removal of RNA after the first strand cDNA synthesis was performed with RNase H (Life Technologies, USA). The cDNA was stored at -20°C until future use. The quantitative real-time PCR (RT-qPCR) was performed in the 96-well plates in a DT-96 Real-time cycler (DNA-Technology, Russia) with 1 μl of cDNA per each qPCR reaction and qPCRmix-HS SYBR (Evrogen, Russia). The thermocycling of each sample was performed in triplicates using the following conditions: 94°C for 3 minutes, followed by 40 cycles of 94°C for 10 s, 60°C for 30 s and 72°C for 30 s. The RealTime_PCR v7.0 software (DNA-Technology, Russia) was used for the data analysis. Expression levels of the NOX genes was calculated relative to glyceraldehyde-3-phosphate dehydrogenase (GAPDH) levels for NIH-3T3 fibroblasts and RPL13a levels for MSC by the comparative ΔC_T_ method. The following primers were used for RT-qPCR: Duox1 (mouse), forward, *CCTACATCAG CCAGGAGAAG ATC*, Duox1 (mouse), reversed, *TGTGCCTCAG TGAACAGTAA GG*, Duox2 (mouse), forward, *AATCAGAAAC ACCACCTTGC G*, Duox2 (mouse), reversed, *ACCACTGCCC TCAAAGTAGT C*, Nox4 (mouse), forward, *GTTTGCATCT TATCCTCCGT CTAG*, Nox4 (mouse), reversed, *TGCTTCTGTG TTTGGTGCAA G*, GAPDH (mouse), forward, *GACCCCTTCA TTGACCTCAA CTAC*, GAPDH (mouse), reverse, *TGGTGGTGCA GGATGCATTG CTGA*; Nox1 (human), forward, *TGTGGGATGA TCGTGACTCC*, Nox1 (human), reverse, *ACGCTTGTTC ATCTGCAATT CC*, Nox2 (human), forward, *CCCTCCTATG ACTTGGAAAT GGA*, Nox2 (human), reverse, *TGGTTTTGAA AGGGTGAGT GAC*, Nox3 (human), forward, *GTTATTTTGG GTTCAACACT GGC*, Nox3 (human), reverse, *CAGCTATCCC ATAGGCGACC*, Nox4 (human), forward, *AGGATCACAG AAGGTTCCAA GC*, Nox4 (human), reverse, *TCCTCATCTC GGTATCTTGC TG*, Nox5 (human), forward, *AGGGCTCTGA GATACTTTTG GAG*, Nox5 (human), reverse, *GCTTTCTTTT CTGGTGCCTG TAC*, Duox1 (human), forward, *GTGCTCCCTC TGTTGTTCGT*, Duox1 (human), reverse, *GCTTCTCAGA CACGATGCTC T*, Duox2 (human), forward, *CCTTCCCTTA GTGAGTCTGC TTC*, Duox2 (human), reverse, *CTGGATGATG ATGGGACTGC TC*, RPL13A (human), forward, *CTCAAGGTCG TGCGTCTGAA*, RPL13A (human), reverse, *ACGTTCTTCT CGGCCTGTTT*.

### Cell migration

NIH-3T3 fibroblasts were grown to confluent in 12-well plates in DMEM containing 10% FBS. Then fibroblasts were deprived in DMEM with 0.1% FBS for 6–8 hours. MSC were grown to confluent in 12-well plates in ASBM containing 10% AdvanceStem Cell Growth Supplement. Then MSC were deprived in ASBM without Supplement for 24 hours. The cell monolayers were scratched with a 1-ml pipette tip 30 min before stimulation with PDGF-BB (final 10 ng/ml) or EGF (final 20 ng/ml), briefly rinsed with the medium and supplied with DMEM containing 0.1% FBS. The inhibitors or vehicle were added 30 min prior to stimulation to final concentrations of 10 μM for LY294002 (PI3-kinase inhibitor), 10 μM for U0126 (Erk1/2 activation inhibitor), 2 mM for apocynin (Nox inhibitor) and 40 U/ml for PEG-conjugated (membrane permeable) catalase. DPI and ebselen were added to 5 μM and 20 μM, respectively, 5 min prior to stimulation to minimize their cytotoxic effects. Then culture plates were transferred on to the microscopic stage of motorized Nikon Ti inverted microscope (Nikon, Japan) equipped with the 4x objective, on-stage culture box, temperature controller set to 37°C and continuous carbogen administration unit. The time-lapse series were continuously acquired every 10 min over 24 h using cooled CCD camera (Nikon, Japan) and the 'Mark and Find' application in NIS Elements (Nikon, Japan) to achieve simultaneous image acquisition in all 12 wells of the plate. This frequency ensures that in each series two successive displacements of a cell are resolved and all cell divisions are captured to be excluded from the analysis later on. The time series were analyzed by manual tracking all cells on the edge of the experimental wounds and measuring their velocity in 2 randomly chosen positions of the wounded areas using free ImageJ software. Routinelly, 50–60 cells were tracked for each data point in one of six independent experiments, accounting for more than 300 cells per point in total.

### Mitotic activity

NIH 3T3 fibroblasts were seeded into 12-well plates in DMEM containing 10% FBS and grown to 10–20% of confluence. Then cells were serum-starved in DMEM containing 0.5% FBS for 12–16 h and the appropriate inhibitors or vehicle were added as indicated in the above section 30 min before stimulation of cells with PDGF-BB (final 10 ng/ml), or EGF (final 20 ng/ml). MSC were grown in 12-well plates to 10–20% of confluence in ASBM containing 10% AdvanceStem Cell Growth Supplement, and deprived in ASBM without Supplement for 24 hours. The time-lapse series were obtained as above using Nikon Ti microscope and cooled CCD camera. The living cells and divisions were counted manually using the ImageJ software. We determined the ratio of total cell divisions over a 24 h period to the number of living cells at the start of recording. We found that mitotic activity greatly depended on the cell density and varied between the particular experiments. Therefore we defined the mitotic activity as the ratio of the values calculated as above to those obtained for the serum-deprived and untreated cells.

### Imaging of cytoplasmic H_2_O_2_

For live imaging of H_2_O_2_ cells were transfected with the HyPer plasmid and serum-starved for 6 hours in MEM (minimal essential medium, Sigma) containing 20 mM HEPES without phenol red and serum. The time-lapse microscopy was performed in the same medium using Leica SP5 (Leica Microsystems GmbH, Germany) equipped with 63x oil immersion objective, the on-stage cell culture box, the temperature controller set to 37°C, and humidifier unit. To excite HyPer fluorescence, the 405 nm diode laser (Coherent, USA) and 488 nm argon laser (Ar-Ion laser LASOS LGK 7872 ML, LASOS Lasertechnik GmbH, Germany) were used. The probe emission was detected every 1 min in a 500–600 nm interval. The excitation intensities were chosen manually to equalize the fluorescence intensity in both channels within the range of 80–150 out of 256 units on the 8 bit grayscale. To minimize phototoxic effects, the least possible light intensity and 800 mV gain on the detector were used. After the first 3–5 images had been acquired, PDGF or EGF were added to final concentrations of 10 and 20 ng/ml, respectively, without stopping the acquisition. The apocynin-treated cells were imaged simultaneously with the untreated cells using the 'Mark and Find' application.

DCF-DA (2’,7’-dichlorofluorescein diacetate) was used for standard measurement of intracellular H_2_O_2_. The 0.2 mM solution of DCF-DA in 96% ethanol was prepared immediately before use and kept in the dark. The cells were seeded into 24-well plates 24 hour prior experiments and serum-deprived overnight. The cell medium was exchanged to phosphate-buffered saline (PBS) and DCF-DA was added to cells at 5 μM final concentration for 30 min. Then the medium was exchanged to DMEM and the cells were stimulated for 25 min. When needed, apocynin was added 10 min before stimulation. After the stimulation the cell medium was exchanged back to PBS and epifluorescence was measured in Nikon Eclipse Ti microscope equiped with 450–490 nm excitation and 515–565 nm emission filters. The autofluorescence of cells and inactive dye was digitally subtracted. The images were processed using free ImageJ software (EMBL ALMF website).

### Cytoplasmic H_2_O_2_ image analysis

The time series were analyzed using the ImageJ software essentially as described [26]. In brief, the images were Gaussian filtered, background subtracted, and converted to 32 bits. The 405-nm stacks were thresholded to remove pixel values from the non-cell background (the Li algorithm, Not-a-Number function). Then the 488-nm stacks were frame-by-frame divided by the corresponding 405-nm stacks. The resulting ratio stack was rendered into pseudocolors using a "Ratio" lookup table. The time course of the HyPer fluorescence was determined in the regions of interest (ROI) within cytoplasm, typically representing not less than 1/10 of the cell area.

### Cell lysis and Western blots

Cells were grown to 70% confluence in 12 well plates with DMEM containing 10% FBS. Prior to stimulation cells were serum-starved for 6 h in DMEM, 0,1% FBS and 1% antibiotic-antimycotic solution (Hyclone, USA). The appropriate inhibitors or vehicle were added 30 min prior to stimulation to final concentrations of 10 μM for LY294002 (PI3-kinase inhibitor), 10 μM for U0126 (Erk1/2 activation inhibitor), 2 mM for apocynin (Nox assembly inhibitor), and 400 U/ml catalase (H_2_O_2_ decomposer). DPI was added 5 min prior to stimulation to 5 μM. The cells stimulated with 10 ng/ml PDGF or 20 ng/ml EGF for 3–60 min before the lysis, depending on the type of experiment. Cell medium was quickly removed at different time points and the plates were transferred on ice. The cells were rinsed with an ice-cold phosphate buffered saline (5.2 mM Na2HPO4, pH 7.4 and 150 mM NaCl) and lysed by 3 x SDS sample buffer (15% β-mercaptoethanol, 6% SDS, 0.2 M Tris, pH 6.8, 40% glycerol and 0.03% bromophenol blue). The residual cells were scraped and the lysates were boiled for 4 min. Each lysate was passed 5 times through a 30-gauge needle with syringe to splinter DNA.

The lysates were resolved in duplicates by 10% SDS-PAGE in the Mini-PROTEAN 3 BioRad Units, in the presence of 0.1% sodium lauryl sulfate (Sigma, USA). The loading amount was controlled by immunostaining for GAPDH, vinculin, total Erk1/2 or total PKB/Akt. The proteins were transferred onto 0.45 μm polyvinylidene fluoride (PVDF) membranes (Amersham, USA) overnight at 150 mA in the buffer containing 25 mM Tris, 0.192 M glycine, pH 8.3, 20% ethanol, 0.02% sodium lauryl sulfate. The membranes were blocked for 1 hour in 5% non-fat milk in TBST (25 mM Tris/HCl, pH 7.4, 150 mM NaCl, 0.1% Tween-20) and cut into horizontal strips according to positions of prestained dual-colored markers (Bio-Rad, USA). This allowed for independent staining of proteins with different molecular weights and avoiding several strippings of one membrane. Thus, each typical set of the western blots hereafter shown in main Figures is obtained from the cell lysates of a typical experiment. Raw images of all the membranes are shown in S5 Fig. The membranes were incubated overnight at 4°C with an appropriate primary antibody in a dilution recommended by the supplier, washed 3 times and incubated for 1 hour at the room temperature with the horseradish peroxidase conjugated secondary antibodies. All blocking procedures, antibody incubation and washings were carried out in TBST (25 mM Tris/HCl, pH 7.4, 150 mM NaCl, 0.1% Tween-20) supplemented with 5% dry nonfat milk, except the primary antibodies to Nox4 and Duox1/2 were used in 1% nonfat milk. The protein bands were visualized by enhanced chemiluminescence (ECL, West Pico, Pierce, USA) using Fusion-SL 3500.WL (Vilber Lourmat, France), or ChemiDoc MP (Bio Rad, USA) imaging systems in their video mode to ensure the linearity of ECL signals.

### Statistics

Statistical analysis was performed using SigmaPlot version 12.5 (Systat Software Inc). All experiments were performed at least three times. Results are shown as mean ± Standard Error of Mean (SEM). The Shapiro-Wilk test was used to test normal distribution. Statistical analysis of two groups was done using Student’s t-test. Multiple comparisons were performed using one-way analysis of variance (ANOVA) and Kruskal-Wallis test (one-way ANOVA on ranks, for not normally distributed data). Post hoc analysis of pairwise comparisons after multiple testing was achieved with Tukey or Dunnett’s tests for one-way ANOVA and with Dunn’s test for ANOVA on ranks. The *p* values < 0.05 were considered statistically significant.

## Results

### PDGF stimulates migration and mitotic activity of mesenchymal cells

**Fig 1:**
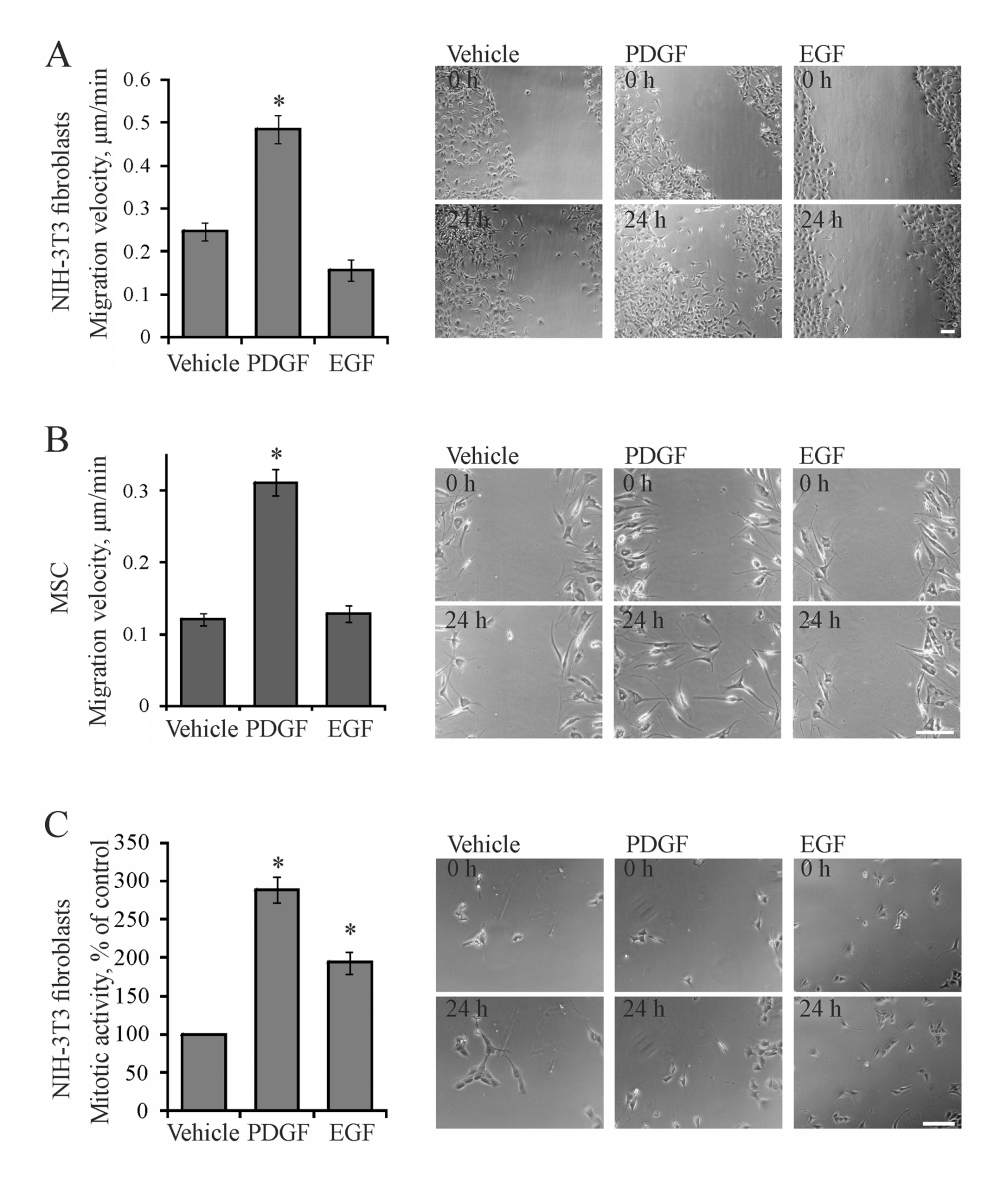
PDGF and EGF effects on mesenchymal cell migration and mitotic activity. (**A**) PDGF, but not EGF stimulates migration of NIH-3T3 fibroblasts in scratch assay; (**B**) PDGF, but not EGF stimulates migration of MSC; (**C**) Both PDGF and EGF stimulate mitotic activity of NIH-3T3 fibroblasts. The 24-hr long time-lapse movies were recorded with 10 min frame intervals. The cell speed was measured by frame-to-frame manual tracking of individual cells; mitotic activity was determined by manual counting of cell divisions. The graphs on the left show mean values ± SE from 6–7 independent experiments; (*) p < 0.05 as compared to vehicle-treated controls. Total 250–340 cells were analyzed for each panel. On the right shown are representative phase contrast images of control cells without stimulation (Vehicle) and cells stimulated with PDGF or EGF at the start (0 h) and the end (24 h) of the typical time-lapse series as indicated. Scale bar, 100 μm.

Becsuse PDGF has been shown to improve directionality of fibroblast movement [6], we sought to establish if it directly accelerates cell locomotion. 3T3 fibroblasts and MSC were subjected to scratch assay and 24 hour long time-lapse movies were recorded. We manually tracked individual cells at the edge of the wounded area, and determined the speed of cell movement. This approach allowed us to exclude the dividing cells from the analysis and quantify the irregular, fibroblast-type movement of individual cells. PDGF increased fibroblast speed nearly twice (Fig 1A) and accelerated the primary MSC migration about 3-fold (Fig 1B).

We measured mitotic activity of fibroblasts by counting number of cell divisions during 24 hours after stimulation of serum-starved cells. PDGF increased it about 3-fold (Fig 1C). This stimulatory effect was strongly inhibited by LY294002, U0126, and apocynin (data not shown), confirming involvement of PI3K, Erk1/2, and ROS. PDGF tended to increase mitotic activity of MSC, however, significant differences were not obtained due to extremely low mitotic activity of fully deprived MSC (1–3 events per microscope field over 24 hours increased to 2–5 by PDGF, data not shown).

### EGF does not stimulate migration of mesenchymal cells

We used a comparative approach to discern PDGF-specific mechanisms of cell migration. We chose EGF as the close relative to PDGF that activates similar signaling pathways. However, even in supraphysiological concentrations EGF had no effect on fibroblast and MSC speed in the scratch assay (Fig 1A and 1B). To confirm that EGF is not a chemoattractant for mesenchymal cells, we titrated the growth factors effects on fibroblast migration. While PDGF increased speed roughly dose-dependently, even two orders of magnitude higher concentrations of EGF failed to accelerate migration (S1 Fig).

To confirm that EGF is functionally active, we determined mitotic activity of EGF-treated fibroblasts. It was increased about 2-fold by EGF as compared to the vehicle-treated cells (Fig 1C). Thus, both PDGF and EGF increased mitotic activity, but only PDGF stimulated migration. This allowed us to use EGF to exclude the irrelevant pathways and dissect migratory signaling by PDGF in mesenchymal cells.

### PDGF-stimulated migration is PI3K- and redox-dependent

PDGF receptors are coupled to PI3K and Erk1/2 pathways [27], as well as to a redox-dependent circuit via generation of intracellular H_2_O_2_ [18,26]. To confirm their contribution to motile responses of 3T3 fibroblasts and MSC to PDGF, we used inhibitory analysis.

**Fig 2:**
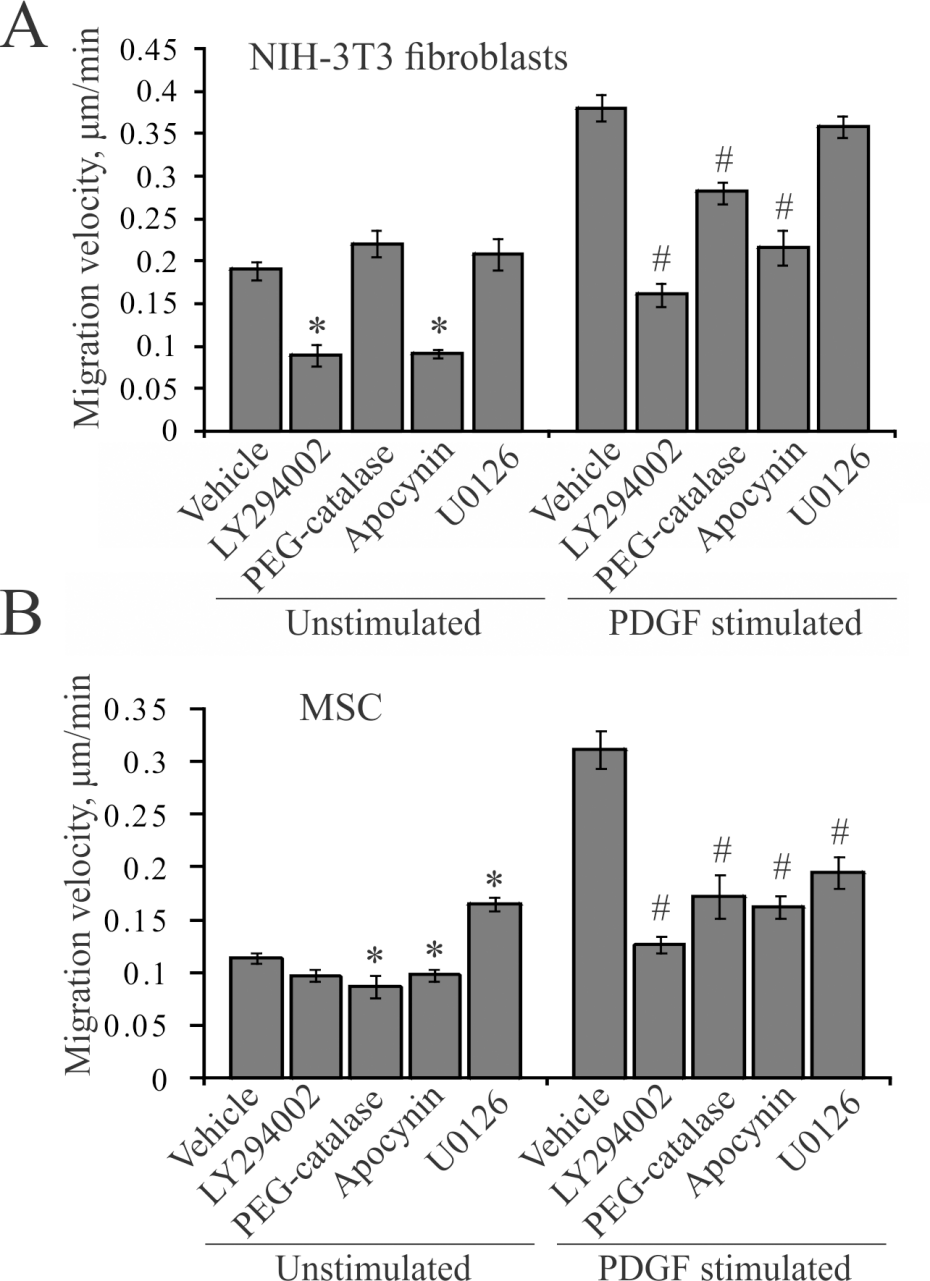
Inhibitory analysis of PDGF-stimulated mesenchymal cell migration. (**A**) Migration of NIH-3T3 fibroblasts. Shown are the mean cell velocities ± SE from 7 independent experiments and 225–340 cells analyzed. (*) p < 0.05 as compared to the unstimulated vehicle control, (#) p < 0.05 as compared to PDGF-stimulated control. (**B**) Migration of MSC. Shown are the mean cell velocities ± SE from 4 independent experiments and 120–200 cells analyzed. (*) p < 0.05 as compared to the unstimulated vehicle control, (#) p < 0.05 as compared to the PDGF-stimulated control.

The specific PI3K inhibitor LY294002 significantly reduced unstimulated and PDGF-stimulated migration of fibroblasts (Fig 2A). LY294002 did not affect basal migration of MSC, but it completely blocked the stimulatory effect of PDGF (Fig 2B). These results indicate that PI3K is required for PDGF-induced migration of mesenchymal cells, which is consistent with the earlier findings [10].

We used several means to inhibit the redox signaling because specific and universal inhibitor is not available. Catalase decomposes hydrogen peroxide that is the ROS end-product [19]. The PEG-conjugate of catalase is cell-permeable, making it useful, albeit a qualitative tool since it is difficult to control its uptake and intracellular activity [28]. PEG-catalase had little, if any effects on the activities of unstimulated cells, suggesting that they are largely redox-independent. However, it inhibited PDGF-stimulated migration of fibroblasts (Fig 2A) and MSC (Fig 2B), indicating that H_2_O_2_ mediates these responses. Because most of the enzymes that generate ROS are flavin-dependent, we used DPI to inhibit their activity. DPI significantly inhibited PDGF-stimulated migration (data not shown), but accurate quantification was not possible because of toxicity of DPI in the long-term assays. Similarly, the general antioxidant ebselen rapidly inhibited migration but also exerted the long-term toxic effects (data not shown). Thus, we used apocynin, an antioxidant and a putative Nox inhibitor, which is used in the millimolar range [26,27]. Apocynin blocked PDGF-induced migration of fibroblasts (Fig 2A) and MSC (Fig 2B), suggesting that ROS, possibly generated by Nox enzymes, are involved. Thus, we concluded that PDGF-induced migration of mesenchymal cells is redox-dependent and likely mediated by H_2_O_2_.

To assess involvement of the Erk1/2 pathway in fibroblast migration we used U0126, a specific inhibitor of Erk1/2 activation. It had no effect on PDGF-stimulated migration of fibroblasts (Fig 2A), but inhibited their mitotic response (data not shown) and PDGF-stimulated migration of MSC (Fig 2B), suggesting that Erk1/2 is involved in migration in cell-type specific manner.

We also verified that PDGF activates PI3K and Erk1/2 in 3T3 cells and the activation inhibitors are selective. PDGF robustly increased phosphorylation of critical activatory sites in PKB/Akt and Erk1/2 (S2 Fig). The corresponding responses were completely and selectively blocked by PI3K inhibitor LY294002 and Erk1/2 pathway inhibitor U0126. This indicates that the lack of the effect of U0126 on PDGF-stimulated migration of fibroblasts is not due to inability of PDGF to activate the Erk1/2 signaling, or of the inhibitor to abrogate this activation. We note that LY294002 reduced phosphorylation of PKB/Akt below the unstimulated (control) level. This is consistent with inhibition of unstimulated migration of fibroblasts by LY294002 (c.f., Fig 2A) and indicates that PI3K pathway is critical for PDGF effects on fibroblast migration.

### PDGF induces sustained accumulation of cytoplasmic H_2_O_2_

**Fig 3:**
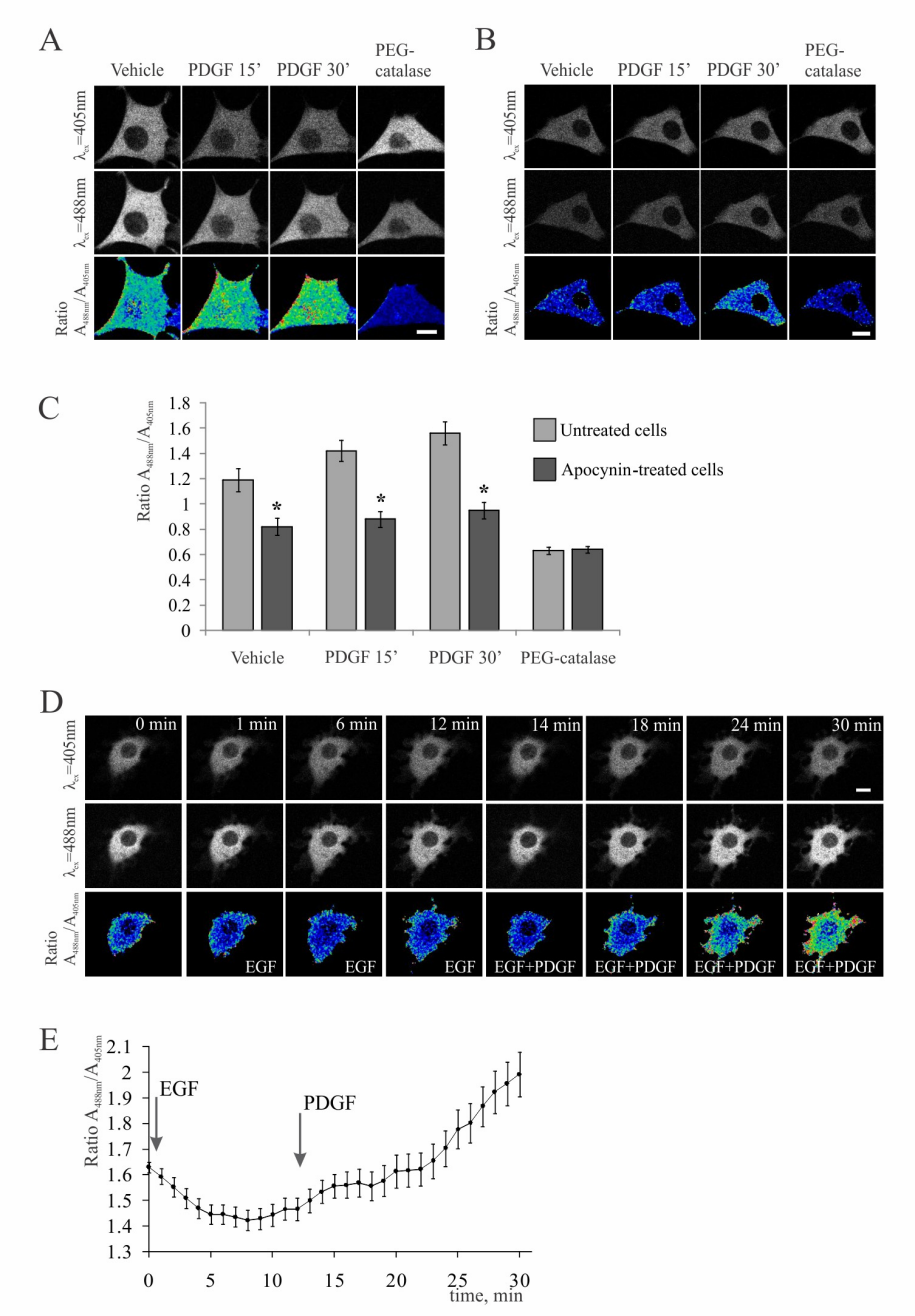
PDGF, but not EGF stimulates accumulation of H_2_O_2_ in fibroblasts. NIH-3T3 fibroblasts were transiently transfected with the plasmid encoding HyPer-NES, the ratiometric cytoplasmic sensor for H_2_O_2_. (**A**) and (**B**) show typical confocal images of the control and apocynin pre-treated cells, respectively, taken at the indicated time points after stimulation with PDGF. The *upper* and *middle* image rows show the changes in fluorescence intensity excited by either 405 nm (top) or 488 nm (middle) lasers. The lower row represents ratio images that report relative changes in cytoplasmic H_2_O_2_. PEG-catalase (40 units/ml) was added 30 min after PDGF to decompose H_2_O_2_ and detect the baseline of the HyPer fluorescense ratio in cytoplasm. Scale bar, 10 μm. (**C**), the time course of H_2_O_2_ accumulation in the control (*grey*) and apocynin treated (*black*) cells. Shown are mean values of the HyPer fluorescence ratio ± SE obtained from 38 control and 65 apocynin-treated cells in 4 independent experiments; (*) p < 0.05 as compared to untreated controls. (**D**)-(**Е**), 3T3 fibroblasts were consequently treated with 20 ng/ml EGF and 10 ng/ml PDGF to directly compare the H_2_O_2_ responses. (**D**) shows confocal images taken at the indicated time points after EGF and PDGF addition depicted underneath. Scale bar, 10 μm. (**E**) shows the time course of H_2_O_2_ accumulation in cytoplasm; addition of growth factor is indicated by arrows. Shown are the mean values of HyPer ratio ± SE from 43 cells analyzed in 3 independent experiments.

To explore the redox response of NIH-3T3 fibroblasts to PDGF we used HyPer, a genetically encoded ratiometric sensor for H_2_O_2_ [26,29]. Because we wanted to follow the H_2_O_2_ responses in cytoplasm, the original sensor was tagged by a short nuclear export sequence (NES) to result in the HyPer-NES, which localized exclusively in cytoplasm. The cells were transiently transfected with the HyPer-NES expressing plasmid and imaged by the time-lapse microscopy to reveal live kinetics of cytoplasmic H_2_O_2_ following PDGF treatment. As shown in Fig 3A, PDGF stimulated sustained increases in fluorescence ratio of the sensor, indicating the elevation of cytoplasmic H_2_O_2_. The H_2_O_2_ response was stable for at least 40 min and immediately disappeared upon subsequent addition of PEG-catalase. Statistics of these results, shown in Fig 3C, demonstrate that PEG-catalase decreased H_2_O_2_ below the level in unstimulated cells. This indicates that a certain basal level of H_2_O_2_ is maintained in untreated cells and it is further increased upon activation by PDGF. Much less changes in HyPer fluorescence were observed in the cells pretreated with apocynin (Fig 3B). Apocynin also reduced basal level of H_2_O_2_ in unstimulated cells, although to the values still higher than those obtained after the application of PEG-catalase. Assuming that the ratio value of HyPer fluorescence in the catalase-treated cells (about 0.6) corresponds to zero level of H_2_O_2_, we calculated that apocynin inhibited the H_2_O_2_ response of the cells to PDGF by about 2.5-fold (Fig 3C). Further translation of the ratio values into absolute concentrations of H_2_O_2_ may not be straightforward and easily performed in the living cells [26,29]. Qualitatively similar results were obtained by the standard DCF staining of unstimulated 3T3 cells and cells treated by PDGF in the absence and presence of apocynin (S3A Fig).

Cytoplasmic H_2_O_2_ was assessed in MSC by DCF staining because the biosensor technique was inadequate due to the low transfection efficiency of these primary cells. The dye accumulated in PDGF-stimulated, but not in unstimulated cells or MSC treated with apocynin (S3B Fig). Although this and previous experiments with 3T3 cells can not unambiguously identify NOX involvement because of the high concentration of apocynin used, they clearly show that PDGF stimulates sustained elevation of H_2_O_2_ in both types of mesenchymal cells.

### PDGF-stimulated phosphorylation of PKB/Akt, but not that of Erk1/2, is redox-dependent

**Fig 4:**
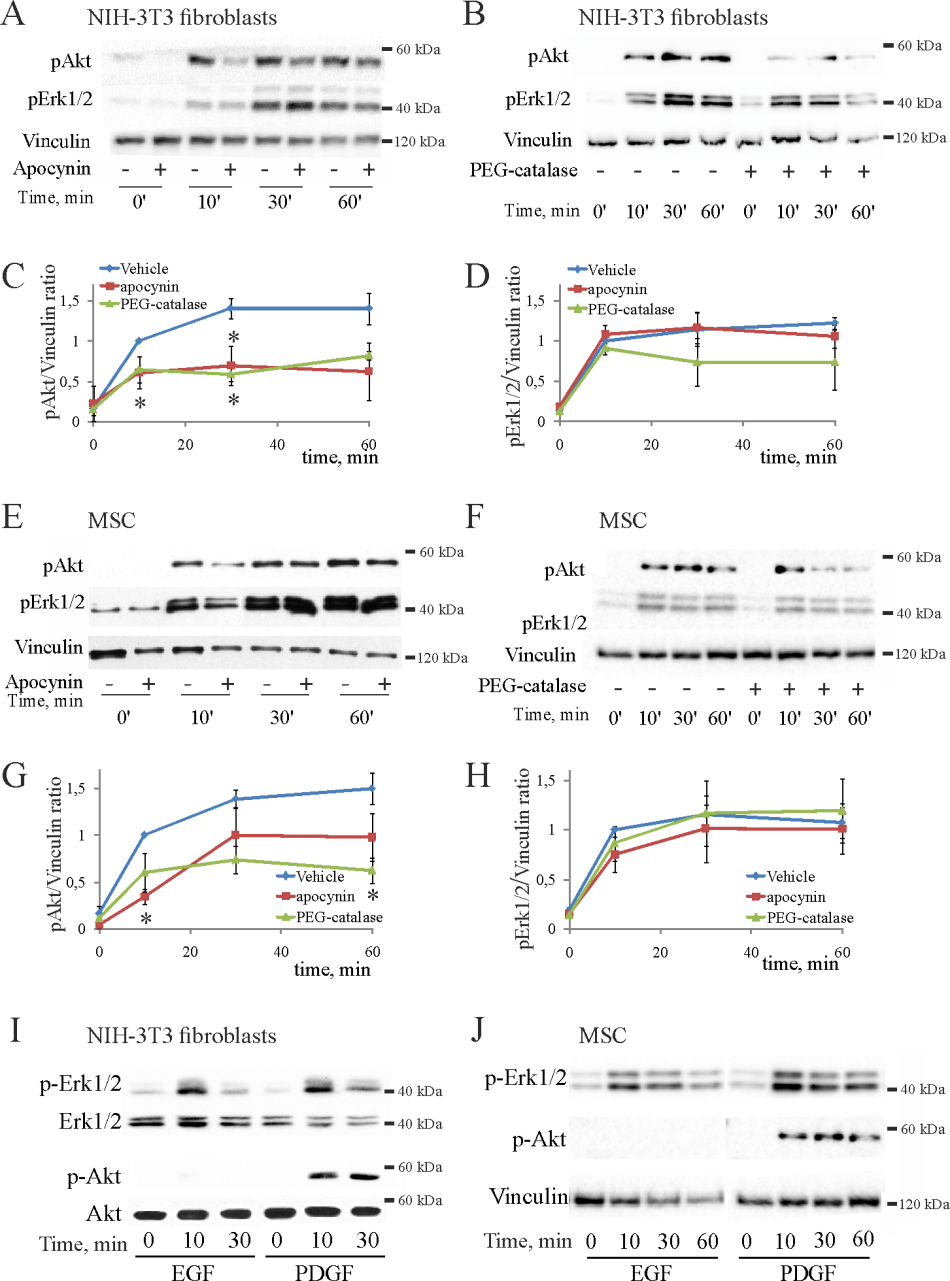
PDGF stimulates redox-sensitive phosphorylation of PKB/Akt in mesenchymal cells, whereas EGF has no effect. (**A**)–(**B**), representative western blots showing the effects of apocynin (**A**) or PEG-catalase (**B**) on phosphorylation kinetics of PKB/Akt and Erk1/2 in PDGF-stimulated 3T3 fibroblasts, and vinculin staining in the same lysates used for the loading control. (**C**)–(**D**), the corresponding changes in phosphorylation of PKB/Akt or Erk1/2 in fibroblasts analyzed in 4 independent experiments by normalization of phosphorylation signals exemplified above to the vinculin content. Additionally, each data set was normalized to the value of 10 min stimulation in uninhibited control, which therefore has no error bar; (*) p < 0.05 as compared to uninhibited controls. (**E**)–(**F**), representative western blots showing the effects of apocynin (**E**) or PEG-catalase (**F**) on phosphorylation kinetics of PKB/Akt and Erk1/2 in PDGF-stimulated MSC, and vinculin staining in the same lysates used for the loading control. (**G**)–(**H**), the corresponding changes in phosphorylation of PKB/Akt or Erk1/2 in MSC analyzed in 4 independent experiments by normalization of phosphorylation signals exemplified above to the vinculin content. As above, each data set was normalized to the value of 10 min stimulation in uninhibited control; (*) p < 0.05 as compared to uninhibited controls. (**I**)–(**J**), PDGF, but not EGF stimulates phosphorylation of PKB/Akt in 3T3 fibroblasts (**I**) and MSC (**J**), but both PDGF and EGF similarly stimulate phosphorylation of Erk1/2. Shown are representative membranes from 2 independent experiments.

Because PI3K, but not Erk1/2, is involved in PDGF-induced migration of 3T3 fibroblasts (c.f. Fig 1A and 1B), which otherwise is redox-dependent, we examined which of the pathways is redox-sensitive. We compared phosphorylation kinetics of PKB/Akt, the PI3K pathway reporter, and those of Erk1/2, in cells treated by PDGF in the absence or presence of apocynin or PEG-catalase (Fig 4A–4H). PDGF stimulated sustained phosphorylation of PKB/Akt and Erk1/2, which plateaued at 30–40 min after stimulation onset and persisted at least for an hour. Apocynin (Fig 4A) and PEG-catalase (Fig 4B) reduced phosphorylation of PKB/Akt in 3T3 fibroblasts, but did not significantly affect phosphorylation kinetics of Erk1/2. The corresponding statistics are shown in Fig 4C and Fig 4D. Similarly, phosphorylation of PKB/Akt, but not that of Erk1/2, was reduced by apocynin (Fig 4E) and PEG-catalase (Fig 4F) in MSC; the corresponding statistics are shown in Fig 4G and Fig 4H. These results indicate that PDGF-stimulated phosphorylation of PKB/Akt is redox-dependent in mesenchymal cell, whereas that of Erk1/2 is not.

### EGF has no effect on cytoplasmic H_2_O_2_ and phosphorylation of PKB/Akt

To test the hypothesis that EGF does not stimulate migration of mesenchymal cells because it can not activate the redox response, we imaged H_2_O_2_ in cells treated with EGF. Indeed, EGF failed to increase cytoplasmic H_2_O_2_ in either 3T3 cells or MSC. First, the standard DCF staining of EGF-treated fibroblasts also revealed no difference from the untreated cells (S4A Fig), and even decreased DCF signal in EGF-treated MSC as compared to corresponding controls (S4B Fig). Second, EGF did not increase cytoplasmic H_2_O_2_ in fibroblasts expressing the HyPer-NES, whereas subsequent addition of PDGF caused marked accumulation of H_2_O_2_ in the same cells (Fig 3D). We note that cells were generally responsive to EGF, which was evident by their intensive ruffling and formation of multiple, irregular membrane protrusions. These protrusions were highly dynamic and disappeared unless stabilized and enlarged by subsequently added PDGF (lower row in Fig 3D). The same results were obtained independently in many cells; the statistics are shown in Fig 3E. They clearly demonstrates that in contrast to PDGF, EGF does not induce the H_2_O_2_ response in fibroblasts.

Because PDGF-induced redox response and migration were associated with increased phosphorylation of PKB/Akt, we compared ability of EGF to activate Erk1/2 and PI3K in 3T3 fibroblasts (Fig 4I) and MSC (Fig 4J). Both growth factors increased phosphorylation of Erk1/2, albeit that induced by EGF was rather transient and returned to the baseline by 30–60 min of stimulation. The PDGF-induced phosphorylation of Erk1/2 also declined, but was sustained over an hour of stimulation (Fig 4I and 4J, upper panels). Thus, both EGF and PDGF similarly activate Erk1/2 signaling in 3T3 fibroblasts and MSC.

In contrast to Erk1/2, phosphorylation of PKB/Akt was stimulated only by PDGF and not altered by EGF both in fibroblasts (Fig 4I) and MSC (Fig 4J). To this end, the obtained results indicated that redox-sensitive activation of PI3-kinase pathway is essential for PDGF-induced mesenchymal cell migration, whereas EGF does not activate redox response, PI3-kinase pathway and migration. The Erk1/2 pathway may not be essential for migration; it is redox-insensitive and similarly activated by both PDGF and EGF.

### NADPH-oxidase expression and silencing in 3T3 fibroblasts and MSC

**Fig 5:**
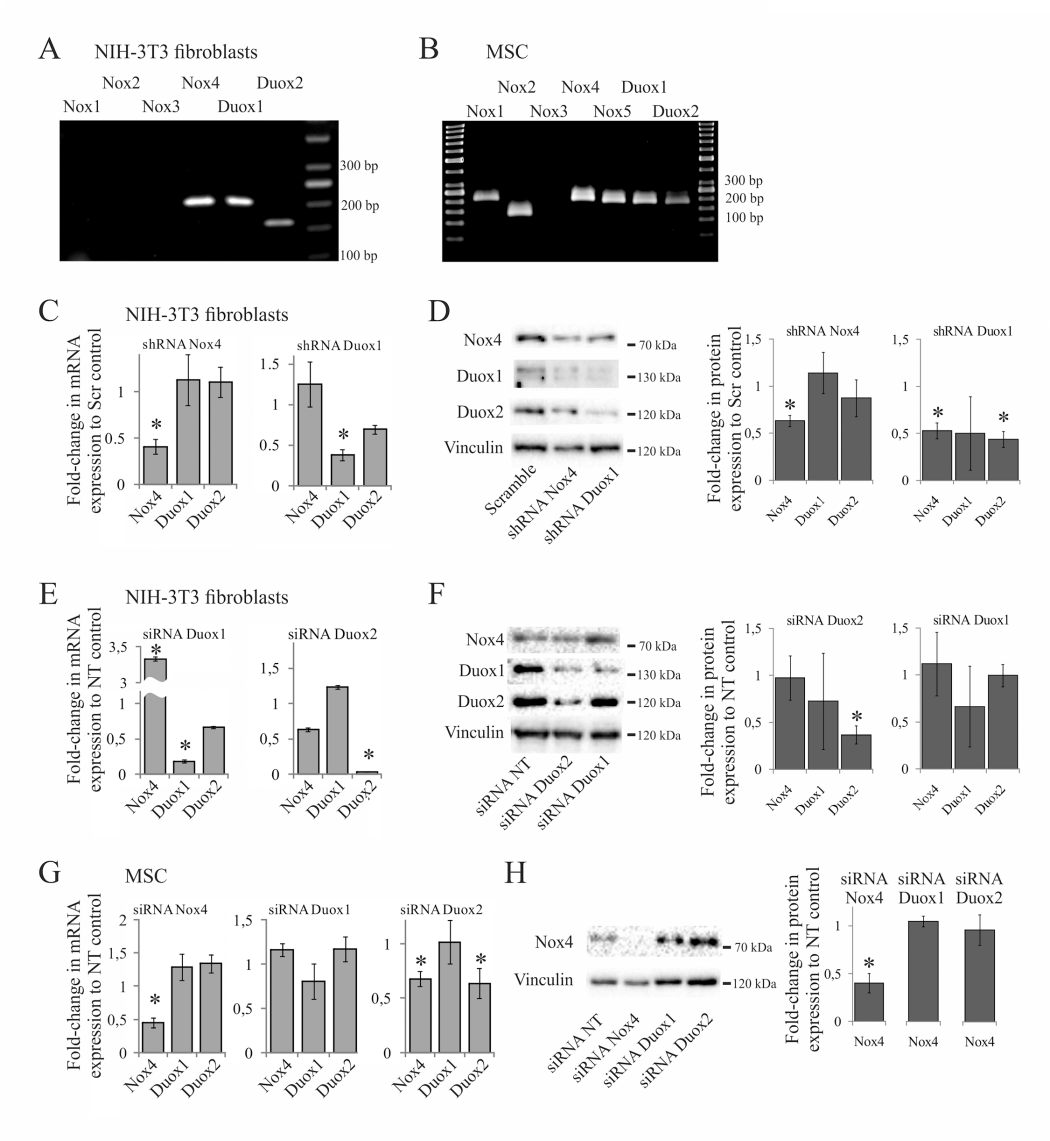
Expression profile and silencing of NADPH-oxidases in mesenchymal cells. (**A**)–(**B**), RT-PCR of NADPH-oxidases in 3T3 fibroblasts and MSC, respectively. Nox5 was not assessed in 3T3 fibroblasts, because it is absent in these cells [30]. (**C**)–(**D**), 3T3 fibroblasts were stably infected by shRNAs to Nox4 or Duox1 and analyzed for corresponding mRNA (**C**) and protein expression (**D**). The graph shows mRNA expression levels normalized to those in cells expressing scrambled shRNA; (*) p < 0.05 as compared to scrambled controls in 3 independent experiments. (**E**)–(**F**), 3T3 fibroblasts were transiently transfected by siRNAs to Duox1 or Duox2 and analyzed for expression of mRNA in 3 independent experiments (**E**) and Nox4 and Duox1/2 proteins in 2 experiments (**F**). The mRNA expression levels were normalized to those in cells treated with non-targeting (NT) siRNA; (*) p < 0.05 as compared to NT controls. The western blots are typical of 2 experiments. (**G**)–(**H**), MSC were transiently transfected by siRNAs to Nox4, Duox1 or Duox2, and analyzed for mRNA in 3 independent experiments (**G**), and Nox4 protein expression in 2 experiments (**H**). The mRNA expression levels were normalized to those in NT controls; (*) p < 0.05 as compared to the NT controls. In this case Duox1/2 protein expression was not significantly altered by corresponding siRNAs (data not shown).

To explore contribution of NADPH-oxidases to activation of PKB/Akt and migration by PDGF we used the si/shRNA-mediated approach. Among five NADPH-oxidases (Nox1-5) and two dual oxidases (Duox1/2) found in vertebrate cells [14,15,19], Nox5 had been reported to be absent in 3T3-fibroblasts [30]. Using real-time PCR we found only Nox4 and Duox1/2 mRNAs expressed in these cells (Fig 5A). Nox1, 2, and 5 were additionally detected in MSC, most likely reflecting the precursor nature of these cells (Fig 5B). We further focused on Nox4 and Duox1/2 as the common sources of signaling H_2_O_2_ in both types of the mesenchymal cells.

To interfere with the expression of Nox4 and Duox1/2 we established sublines of 3T3 fibroblasts that stably express shRNA to Nox4 or Duox1. Expression of the targeted mRNAs (Fig 5C) and proteins (Fig 5D) in these cells was analyzed by quantitative RT-PCR and western blots, respectively. Although the Nox4 mRNA was significantly reduced in the cells expressing Nox4 shRNA (Fig 5C), the Nox4 protein level was not sufficiently affected (Fig 5D, lane 2 shRNA Nox4, as compared to control lane 1 and vinculin as the loading control). Therefore these cells were excluded from further analysis. On the contrary, the Duox1 shRNA decreased expression of both Duox1 and Duox2 mRNAs, as well as the proteins, while did not dramatically affect that of Nox4 (Fig 5D, lane 3, siRNA Duox1). Thus, we designated the 3T3 cells expressing the Duox1 shRNA as those having reduced Duox1/2 expression, and used them in further analysis.

In complementary experiments we targeted Duox1/2 expression in fibroblasts by corresponding siRNAs (Fig 5E and 5F). At the messenger level, each of Duox1 or Duox2 siRNAs significantly decreased expression of corresponding mRNA, but also tended to reduce expression of each other mRNA (Fig 5E). Thus, siRNA to Duox1 significantly silenced the Duox1 mRNA, but also to some extent reduced the Duox2 mRNA (Fig 5E, the graph on the left). Similarly, siRNA to Duox2 significantly silenced the Duox2 mRNA and tended to reduce the Duox1 mRNA (Fig 5E, the graph on the right). At the protein level only Duox1 siRNAs selectively silenced Duox1 (Fig 5F, lane 3 siRNA Duox1), while Duox2 siRNAs reduced both the Duox1 and Duox2 proteins (Fig 5F, lane 2 siRNA Duox2). In neither cells expression of Nox4 protein was drastically affected (Fig 5F, upper row), despite it was altered at the mRNA level (Fig 5E, the Nox4 bars). Thus, we concluded that Duox1 siRNAs selectively silence Duox1 expression, whereas Duox2 siRNAs simultaneously silence both Duox1 and Duox2 expression in 3T3 fibroblasts.

The progenitor properties of MSC can not be maintained during the long-term passaging, which precludes generation of the stable cell lines expressing shRNAs. Therefore we used siRNA-mediated silencing to selectively reduce the expression of NADPH-oxidases in MSC (Fig 5G). Only Nox4 expression was silenced selectively at the mRNA level (Fig 5G, the left graph) and at the protein level (Fig 5H), whereas siRNAs to Duox1 were ineffective (Fig 5G, the graph in the center), and siRNAs to Duox2 were non-specific reducing both the Duox2 and Nox4 mRNAs (Fig 5G, the right graph). Whether Duox1 or Duox2 siRNAs reduce expression of corresponding proteins has not been studied and only the Nox4-deficient MSC were selected for further analysis along with the control cells treated by the non-targeting (NT) siRNAs.

### Duox1/2 and Nox4 mediate redox control of PDGF-induced phosphorylation of PKB/Akt and migration

**Fig 6:**
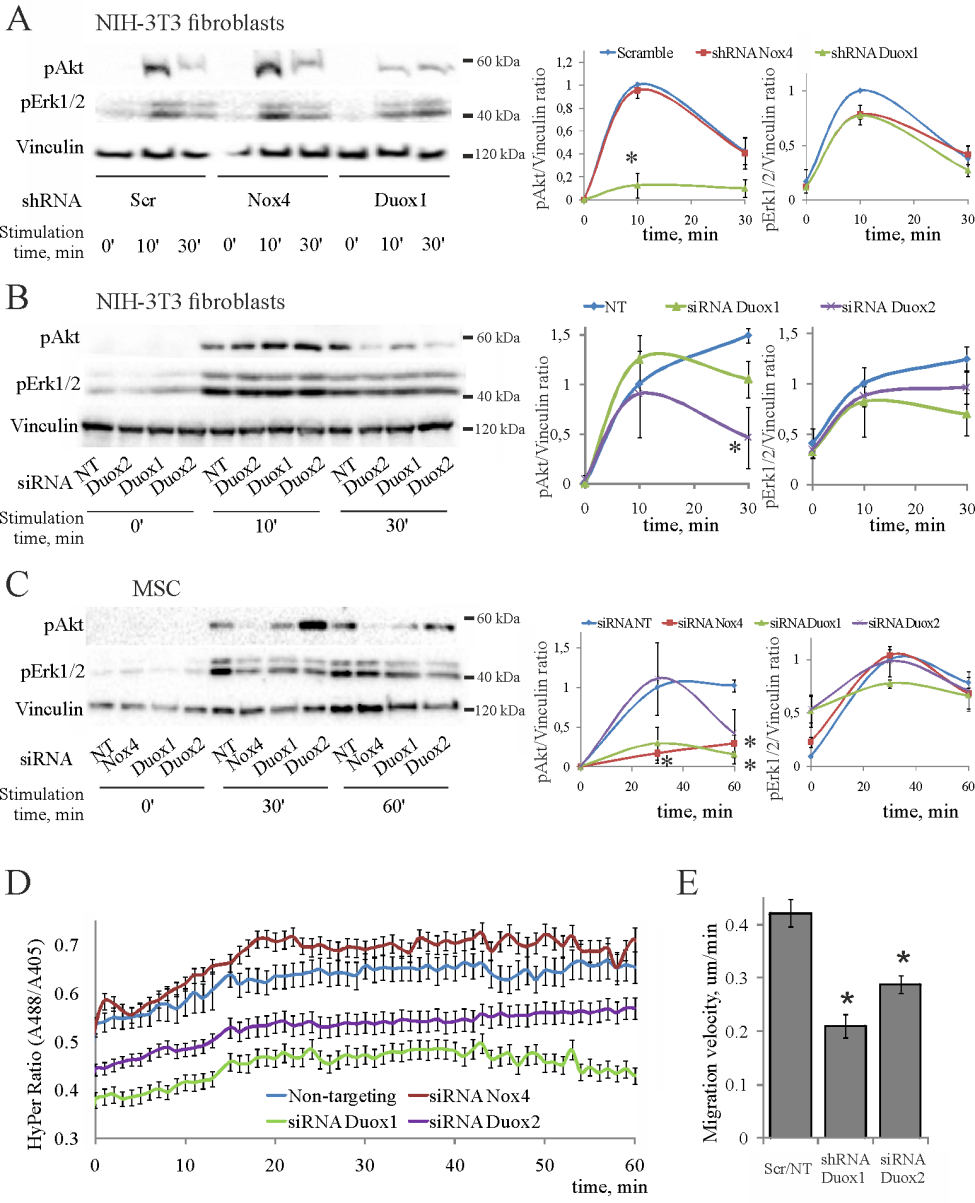
Duox1/2 and Nox4 mediate the PDGF-induced responses in mesenchymal cells. Representative blots are shown on the left, statistics are shown on the right. The data are normalized to 10 min of stimulation in scrambled/NT controls for fibroblasts and 30 min of stimulation for MSC, which have therefore no error bars; (*) p < 0.05 as compared to scrambe (*scr*) or non-targeting (*NT*) controls from 2–4 independent experiments. (**A**) PDGF-induced phosphorylation of PKB/Akt and Erk1/2 in 3T3 fibroblasts stably expressing the indicated shRNAs. (**B**) PDGF-induced phosphorylation of PKB/Akt and Erk1/2 in 3T3 fibroblasts transiently transfected by indicated siRNAs. (**C**) PDGF-induced phosphorylation of PKB/Akt and Erk1/2 in MSC transiently transfected by indicated siRNAs. (**D**) Kinetics of cytoplasmic H_2_O_2_ accumulation in 3T3 fibroblasts pre-treated by indicated siRNAs; PDGF added at 0 min. (**E**) Effects of Duox1/2 silencing on speed of 3T3 fibroblast migration. Shown are the results of shRNA- and siRNA-mediated silencing of Duox1/2 compared, respectively, to scramble and NT controls, which moved with identical speeds.

We assessed phosphorylation of PKB/Akt and Erk1/2 in PDGF-treated fibroblasts that stably express shRNA to Nox4 or Duox1/2 (Fig 6A). The phosphorylation response was not altered in cells expressing Nox4 shRNA, which is consistent with unaffected expression of Nox4 protein (c.f. Fig 5D). Thus, whether Nox4 is involved remains undetermined. In contrast, PDGF failed to stimulate phosphorylation of PKB/Akt in cells with reduced expression of Duox1/2 as compared to PKB/Akt phosphorylation in the scrambled controls, whereas it stimulated phosphorylation of Erk1/2 in these cells. This indicates that Duox1/2 control phosphorylation of PKB/Akt, but not that of Erk1/2 in 3T3 fibroblasts.

To get further insight to which isoform of Duox1/2 in fibroblasts is involved, we used siRNA approach. We confirmed that phosphorylation of PKB/Akt at 30 min of PDGF stimulation was lower significantly in cells treated by Duox2 siRNAs (Fig 6B) that reduce expression of both Duox1 and Duox2 (c.f. Fig 5F). In contrast, selective silencing of Duox1 had no effect (Fig 6B), suggesting that this is Duox2 that augments PDGF-induced phosphorylation of PKB/Akt in 3T3 fibroblasts. As expected, the activation of Erk1/2 was not affected in either case.

Because the above results do not report on the potential role of Nox4 in phosphorylation of PKB/Akt, we determined the effects of selective Nox4 knockdown in MSC. As shown in Fig 6C, PDGF-induced phosphorylation of PKB/Akt was reduced in MSC treated by the siRNAs that target Nox4 or Duox1. However, only silencing of Nox4 mRNA and protein was significant in the same cells (see Fig 5G and 5H), indicating that Nox4 controls phosphorylation of PKB/Akt in MSC. Perhaps because Duox2 siRNAs non-specifically reduced both Nox4 and Duox2 mRNAs (Fig 5G), the phosphorylation of Akt was also affected by Duox2 siRNAs (Fig 6C), further suggesting that Nox4 is involved. Whether Duox1/2 control phosphorylation of PKB/Akt remains unclear and needs further studies. Taken together, the results obtained with two types of mesenchymal cells suggest that both Nox4 and Duox1/2 are involved, in cell-specific manner, in the redox control of PDGF-stimulated phosphorylation of PKB/Akt.

To explore which NADPH-oxidase mediates PDGF-induced accumulation of cytoplasmic H_2_O_2_, we transfected 3T3 fibroblasts that express HyPer-NES with siRNAs to Nox4 and Duox1/2. The kinetics in the sensor fluorescence ratio following PDGF addition are shown in Fig 6D. They demonstrate lower H_2_O_2_ signals in 3T3 fibroblasts treated with siRNAs to Duox1 or 2, but not to Nox4. This confirms that Duox1/2, but not Nox4 mediates redox responses of 3T3 cells to PDGF.

Finally, we ascertained that Duox1/2 mediate PDGF-induced migration of fibroblasts. We measured speed of migration of 3T3 cells transfected with Duox1/2 siRNAs and found it is approximately twice reduced as compared to that of the control cells treated with the non-targeting siRNA (Fig 6E). Taken together, these results indicate that Duox1/2 mediate redox control of PKB/Akt phosphorylation and fibroblast migration stimulated by PDGF, while Nox4 is involved in the redox control of PKB/Akt phosphorylation in MSC.

## Discussion

We report that PDGF-stimulated migration of mouse 3T3 fibroblasts and primary human MSC ultimately involves sustained accumulation of cytoplasmic H_2_O_2_, redox-dependent activation of PI3-kinase and phosphorylation of PKB/Akt. Nox4 in MSC and Duox1/2 in 3T3 fibroblasts have been identified as responsible for the redox responses to PDGF. The critical contribution of the redox component was corroborated by the comparative studies, which showed that EGF failed to increase cytoplasmic H_2_O_2_, activate PKB/Akt and stimulate migration in mesenchymal cells. Furthermore, we identified the PI3-kinase pathway to be specifically redox regulated as compared to the Erk1/2 pathway, which was activated similarly by PDGF and EGF in a redox-insensitive fashion. These results are consistent with the idea that redox-dependent phosphorylation and activation of PKB/Akt, but not that of Erk1/2, mediates stimulatory effect of PDGF on mesenchymal cell migration.

PDGF is the major chemoattractant and mitogen for fibroblasts, whereas EGF targets epithelial cells. EGF has been also shown to stimulate migration of some fibroblasts [31], perhaps as a result of variations in the cell type, culturing and/or migration protocols. In our case 3T3 fibroblasts were responsive to EGF as evident by their mitotic response (Fig 1C) and extensive membrane ruffling induced by EGF (lower images in Fig 3D). Perhaps, this unlocalized membrane activity prevented stabilization of single protrusions and led to the loss of directional persistence of movement in the latter case. This might be the reason why EGF even decreased the average velocity of these cells (Fig 1B). While the full mechanistic details of the different cell responses to PDGF and EGF were out of scope of this study, we used this feature to single out signaling events relevant to migration as those induced by PDGF and not by EGF. Thus, we found that PDGF increases intracellular H_2_O_2_ and redox-dependent phosphorylation of PKB/Akt, whereas EGF does not. Therefore, redox activation of PKB/Akt appears to be critical for PDGF-activated migration of mesenchymal cells.

Similarly, the Erk1/2 pathway is known to control cell cycle progression [8] and integrin-mediated motility [32] downstream of PDGF and EGF receptors [27]. We confirmed that Erk1/2 control mitotic activity of fibroblasts (data not shown) and become activated by both PDGF and EGF in mesenchymal cells (Fig 4I and 4J). This activation was also required for PDGF-induced migration of MSC (Fig 2B), but was dispensable for that of 3T3 fibroblasts (Fig 2A). The migration-related targets of Erk1/2 are likely located in cytoplasm and cell adhesions [32]. Because chemotactic signaling pathways are highly redundant in cells (reviewed in [33]), it is possible that requirement for Erk1/2 is cell-type specific and can be supplanted by other means. In addition, Erk1/2 translocates into nucleus where it phosphorylates and activates a number of transcription factors and early genes to mediate proliferation responses in non-redundant manner [34]. This may explain why the mitotic activity of fibroblasts is Erk1/2-dependent, whereas migration is not.

The PDGF-stimulated migration and mitotic activity of fibroblasts and MSC involves PI3-kinase pathway. This is consistent with observations that PI3K controls migration, proliferation and survival in many cell types including fibroblasts [6,11,33]. PKB/Akt is thought to mediate the PI3K signaling to cell migration [10]. It is coupled to chemotactic receptors in Dictyostelium cells [35] and mediates VEGF-stmulated migration of mammalian endothelial cells [36]. In mammalian fibroblasts and epithelial cancer cells it acts, in isoform-specific manner, downstream of Rac and Cdc42 at the leading edge [37–40].

Redox activation of PKB/Akt via H_2_O_2_ has been also reported [41–43], but how it is associated with cell migration is still uncertain. The reversible oxidation of Akt2 isoform in mouse embryo fibroblasts has been suggested to lead to increased migration and G1>S cell cycle transition without affecting phosphorylation of PKB/Akt at the activating Thr-308 [44]. We show that PDGF stimulates redox-sensitive phosphorylation of Ser-473 in PKB/Akt (Fig 4), which is also needed for full activation of the kinase [45]. Because phosphorylation of Ser-473 is mediated by mechanistic target of rapamycin complex 2 (mTORC2) [46], the possibility arises that mTORC2 activity may be also redox-sensitive, similar to that reported for mTORC1 [47]. Rictor is critical for mTORC2 assembly, and cell migration mediated by RhoGDI [48]. Whether it mediates redox activation of mesenchymal cell migration, remains for the future studies.

Several lines of evidence implicate intracellular ROS and NADPH-oxidases in cell migration [49–51]. NADPH-oxidases generate free radicals and H_2_O_2_ in receptor-dependent fashion [14]. Relatively stable H_2_O_2_ regulates activity of signaling kinases and phosphatases through reversible oxidation of their essential cysteines [16,17] and rapidly gains a second messenger status [15,19,21]. Using shRNA- and siRNA-mediated analyses we provide evidence for the involvement of Duox1/2 and Nox4 in the redox control of migration of fibroblasts and their precursor cells mediated by PKB/Akt in response to PDGF (Figs 5 and 6). These results are consistent with the studies on the immune cells, which showed that Duox1/2 is responsible for the large-scale gradients of H_2_O_2_ that navigate leukocytes into wounds in Zebrafish animal model [22,23], and further suggest that it may be the case for mammalian fibroblasts. The mechanism that couples the Duox enzymes to PDGF receptors in fibroblasts may involve rises in intracellular Ca^2+^ [15] and needs further studies.

It remains presently unclear how Nox4 is coupled to PDGF activation in MSC because it lacks the Rac-binding site and its activity is thought to be largely receptor-independent [14,15]. However, Nox4 is similarly involved in insulin-like growth factor-I induced migration of vascular smooth muscle cells [52]. Perhaps, the mechanism may involve the p22phox binding partner Poldip2a that was described in vascular smooth muscle cells [53], or a yet unknown endocytic signaling by iternalized receptors, because H_2_O_2_-generating activity is associated with endosomes [26] and Nox4 has been localized to endoplasmic compartment [19].

Finally, using live imaging of intracellular H_2_O_2_, we found that kinetics of its accumulation in cytoplasm are comparable to those of PKB/Akt phosphorylation in response to PDGF (Fig 3). Based on the proposed mechanisms of H_2_O_2_ action [16,17], we suggest that H_2_O_2_ acts to maintain an increased activity of PKB/Akt over the period required for fibroblast to stabilize pseudopod growth at the cell front [54]. This mechanism has been shown to underlie the persistent motility of such amoeboid cell as *Dictyostelium* and neutrophils [55]. It may also apply to mesenchymal cells that exhibit slow dynamics and usually require 40–60 min to grow a pseudopod [56]. The redox signaling may help to convert the transient activation of PI3K, which was detected by imaging of phosphatidylinositol (3,4,5)-trisphosphate (PIP_3_) in these cells [9], to persistent activation of PKB/Akt and its cytoskeletal targets that would be sufficient to span the pseudopod cycle and couple the growth of two successive protrusions at the leading edge.

In conclusion, we showed that PDGF-stimulated migration of mesenchymal cells is redox-dependent; the PI3-kinase/PKB/Akt pathway appears to be a target. This implicates the receptor-dependent redox mechanisms as important constituents of the stromal cell biology, in addition to that recently observed for the circulatory leukocytes. NADPH-oxidases emerge as potential targets to manipulate migratory and/or proliferative activity of stromal fibroblasts during tissue healing and regeneration.

## Supporting Information

S1 Fig Dose-dependence of growth factor-induced 3T3 fibroblast migration. The cells were stimulated by indicated concentrations of PDGF and EGF and their migration speed was determined by manual tracking as decribed in the Methods section. (*) p < 0.01 as compared to unstimulated control.
(TIFF)

S2 Fig Selectivity of the inhibitors of PI3-kinase and Erk1/2 activation in PDGF-stimulated NIH-3T3 fibroblasts. Cell lysates were collected 10 min after addition of 10 ng/ml PDGF or vehicle (control) and analyzed by western blots. Representative membranes from 2 independent experiments are shown, the irrelevant lanes are cut inbetween the control (vehicle) and other lanes of the same membrane. The inhibitors were added 30 min prior PDGF to 10 μM final concentration.
(TIFF)

S3 Fig PDGF stimulates apocynin-sensitive ROS production in mesenchymal cells. 3T3 fibroblasts (A) or MSC (B) were treated for 20 min with 10 ng/ml PDGF in the presence or absence of apocynin as indicated. *Left*, representative images; scale bar, 50 μm. Right, normalized DCF epifluorescence ± SE from 3 independent experiments; (*) p < 0.05 as compared to the vehicle controls.
(TIFF)

S4 Fig EGF does not increase ROS in mesenchymal cells. 3T3 fibroblasts (A) or MSC (B) were treated for 20 min with 20 ng/ml EGF. *Left*, representative images; scale bar, 50 μm. Right, normalized DCF epifluorescence ± SE from 3 independent experiments; (*) p < 0.05 as compared to the vehicle control.
(TIFF)

S5 Fig Raw data for Figs 4–6. Shown are ECL images of Western blot membranes used to generate Figs 4A, 4B, 4E, 4I, 5D, 5F, 5H, 6A, 6B and 6C, as indicated on the top of each page. The breakdown of sample loading in shown for each gel in tables underneath.
(PDF)
