# Supplementary figures and images for: Nox4 and Duox1/2 Mediate Redox Activation of Mesenchymal Cell Migration by PDGF
Source: PLoS One. 2016 Apr 25;11(4):e0154157. doi: 10.1371/journal.pone.0154157 (PMC4844135; doi:10.1371/journal.pone.0154157)

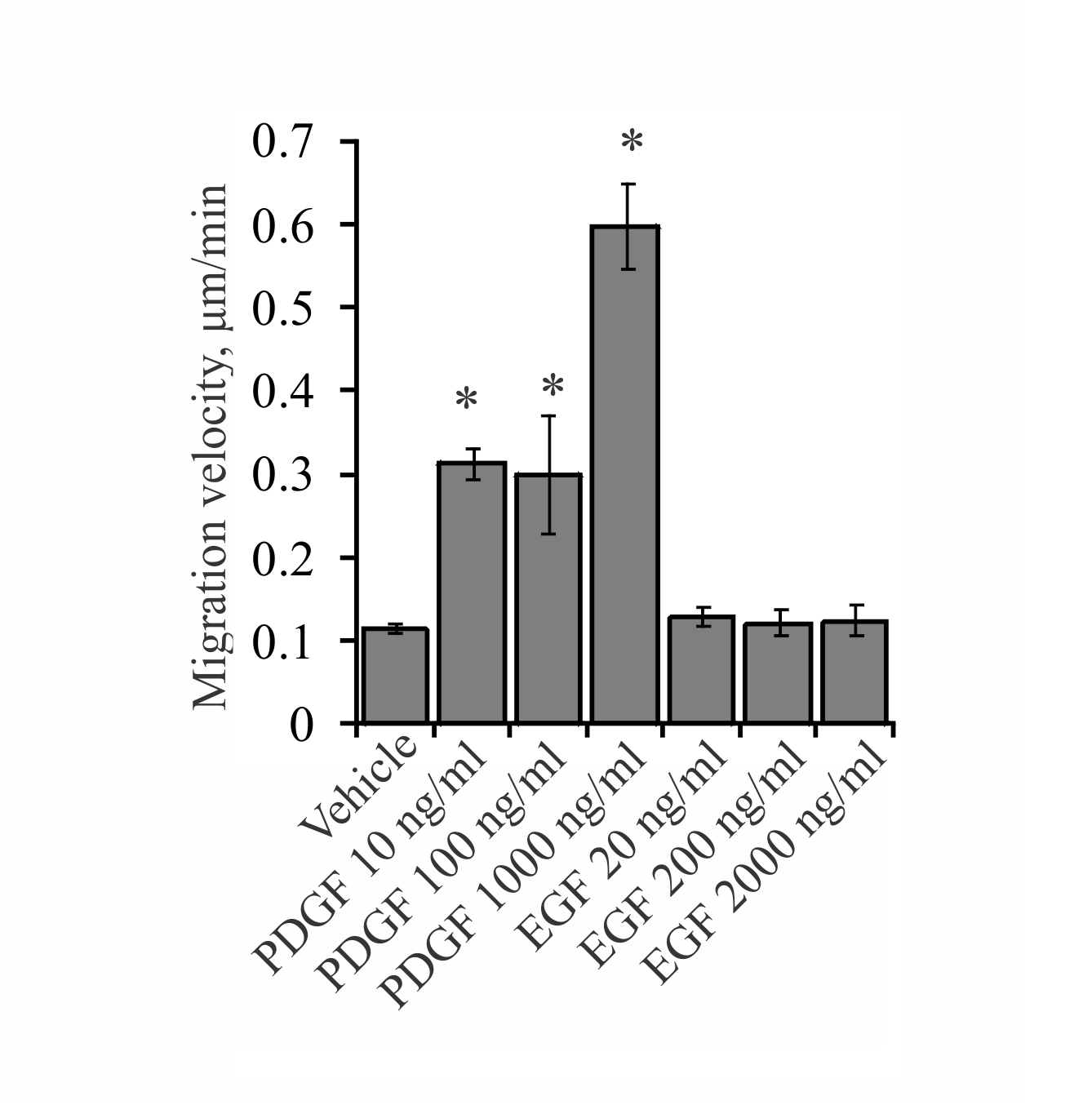

Supplement: S1 Fig — The cells were stimulated by indicated concentrations of PDGF and EGF and their migration speed was determined by manual tracking as decribed in the Methods section. (*) p < 0.01 as compared to unstimulated control. (TIFF) [file pone.0154157.s001.tiff]

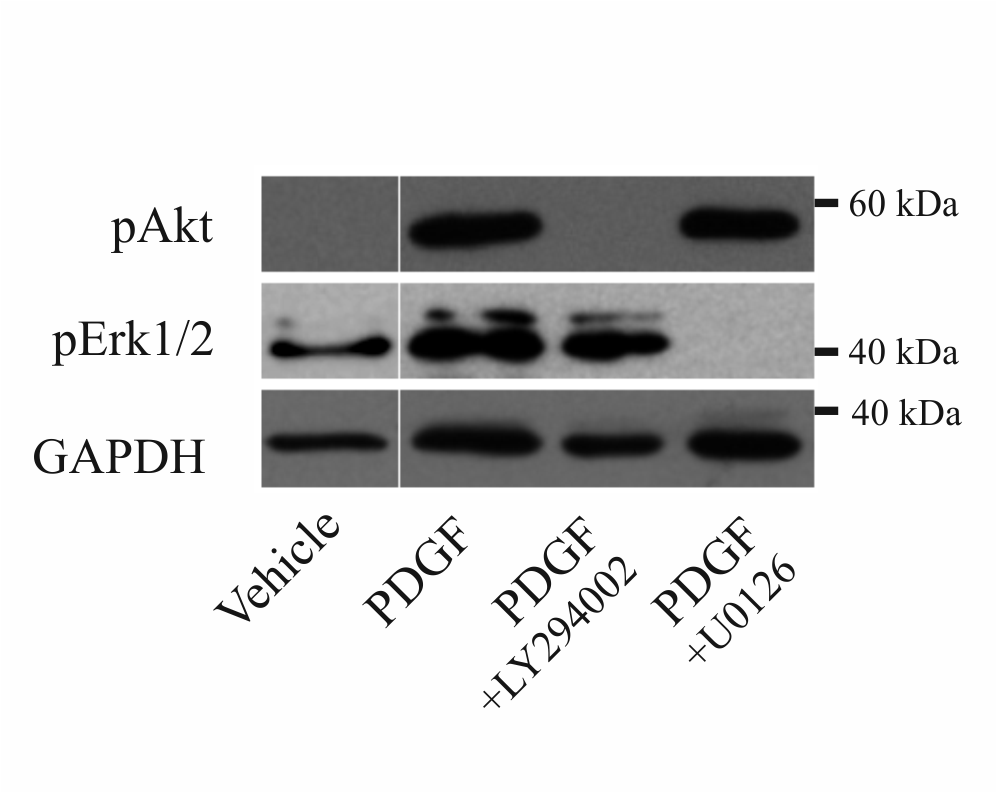

Supplement: S2 Fig — Cell lysates were collected 10 min after addition of 10 ng/ml PDGF or vehicle (control) and analyzed by western blots. Representative membranes from 2 independent experiments are shown, the irrelevant lanes are cut inbetween the control (vehicle) and other lanes of the same membrane. The inhibitors were added 30 min prior PDGF to 10 μM final concentration. (TIFF) [file pone.0154157.s002.tiff]

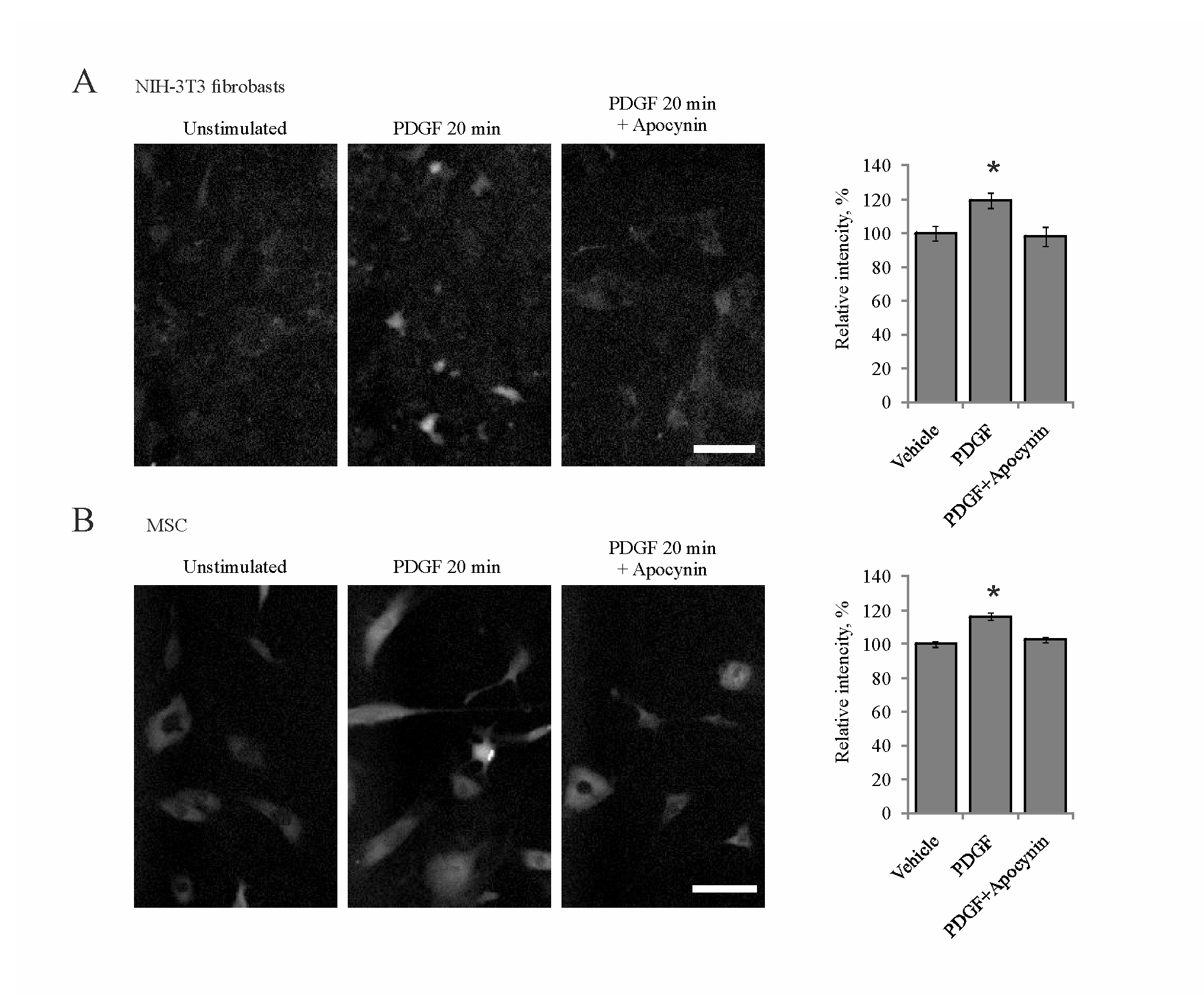

Supplement: S3 Fig — 3T3 fibroblasts (A) or MSC (B) were treated for 20 min with 10 ng/ml PDGF in the presence or absence of apocynin as indicated. Left, representative images; scale bar, 50 μm. Right, normalized DCF epifluorescence ± SE from 3 independent experiments; (*) p < 0.05 as compared to the vehicle controls. (TIFF) [file pone.0154157.s003.tiff]

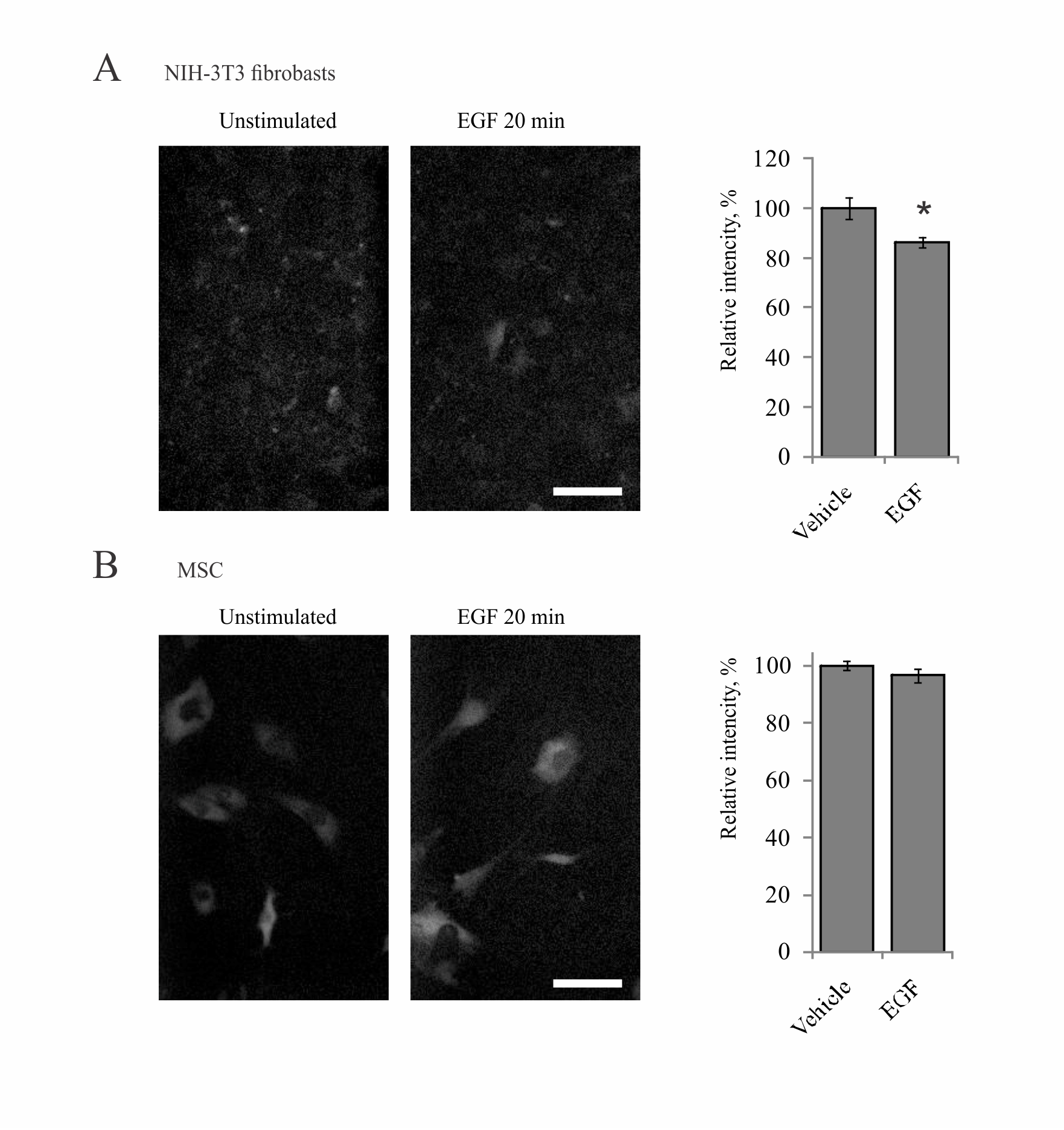

Supplement: S4 Fig — 3T3 fibroblasts (A) or MSC (B) were treated for 20 min with 20 ng/ml EGF. Left, representative images; scale bar, 50 μm. Right, normalized DCF epifluorescence ± SE from 3 independent experiments; (*) p < 0.05 as compared to the vehicle control. (TIFF) [file pone.0154157.s004.tiff]
