# Supplementary material for: Nox4 and Duox1/2 Mediate Redox Activation of Mesenchymal Cell Migration by PDGF
Source: PLoS One. 2016 Apr 25;11(4):e0154157. doi: 10.1371/journal.pone.0154157 (PMC4844135; doi:10.1371/journal.pone.0154157)
Supplement: S5 Fig — Shown are ECL images of Western blot membranes used to generate Figs 4A, 4B, 4E, 4I, 5D, 5F, 5H, 6A, 6B and 6C, as indicated on the top of each page. The breakdown of sample loading in shown for each gel in tables underneath. (PDF) [file pone.0154157.s005.pdf]

Raw images for Figs.4A

Fig 4A (1)

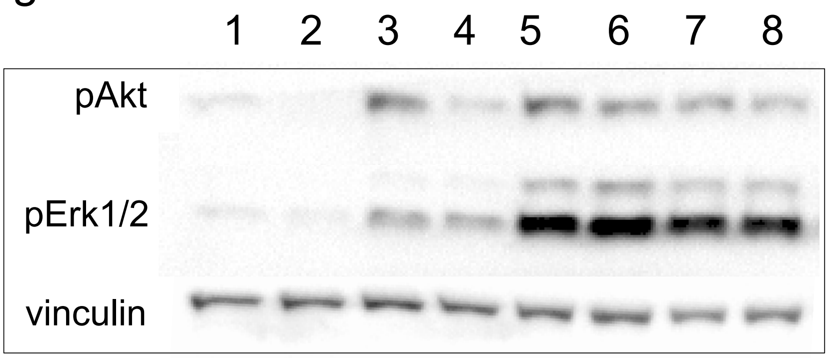

Fig 4A (2)

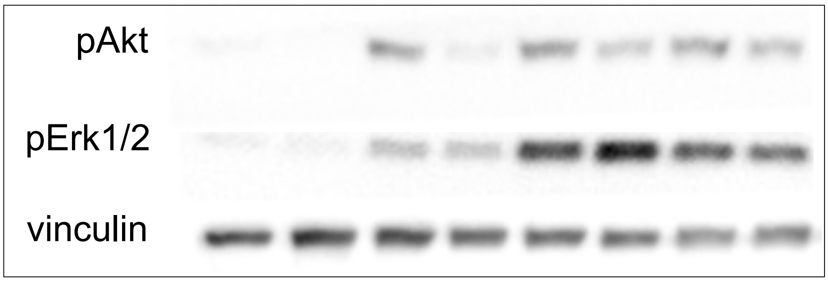

Gel 4A (1)

Lane #	Description
1	No addition
2	Apo
3	10' PDGF
4	10' PDGF+Apo
5	30' PDGF
6	30' PDGF+Apo
7	60' PDGF
8	60' PDGF+Apo

Gel 4A (2)

Lane #	Description
1	No addition
2	Apo
3	10' PDGF
4	10' PDGF+Apo
5	30' PDGF
6	30' PDGF+Apo
7	60' PDGF
8	60' PDGF+Apo

Raw images for Figs.4A (continued) and Fig.4B

Fig 4A (3)
and
Fig 4B (1)

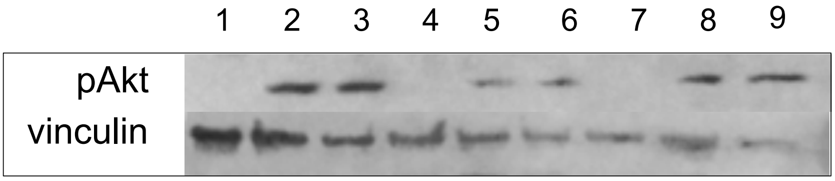

Fig 4A (4)
and
Fig 4B (2)

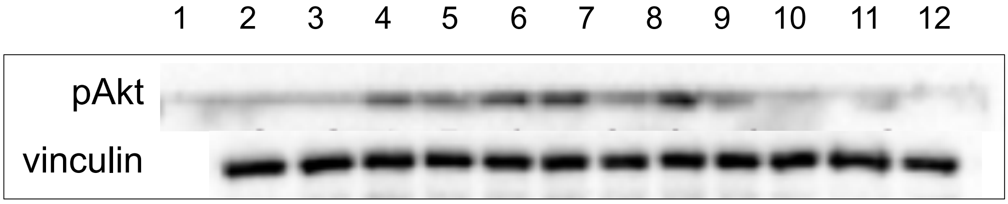

Gel 4A (4) / 4B (1)

Lane #	Description
1	No addition
2	10’ PDGF
3	30’ PDGF
4	Apo
5	10’ PDGF+Apo
6	30’ PDGF+Apo
7	Cat
8	10’ PDGF+Cat
9	30’ PDGF+Cat

Gel 4A (5) / 4B (2)

Lane #	Description
1	No addition
2	Apo
3	Cat
4	10’ PDGF
5	10’ PDGF+Apo
6	10’ PDGF+Cat
7	30’ PDGF
8	30’ PDGF+Apo
9	30’ PDGF+Cat
10	60’ PDGF
11	60’ PDGF+Apo
12	60’ PDGF+Cat

Raw images for Fig.4B (continued)

Fig 4B (3)

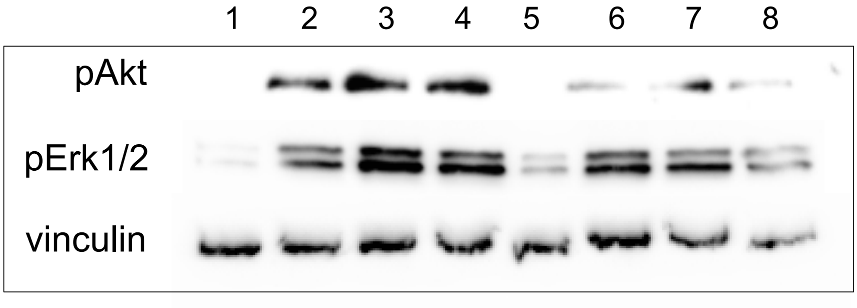

Fig 4B (4)

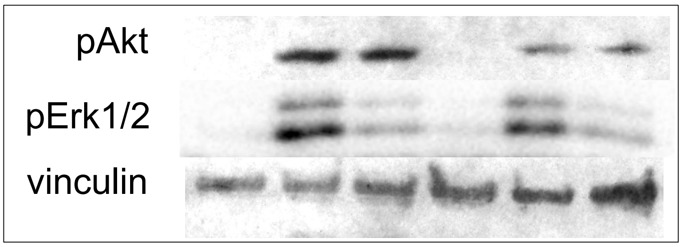

Gel 4B (3)

Lane #	Description
1	No addition
2	10’ PDGF
3	30’ PDGF
4	60’ PDGF
5	Cat
6	10’ PDGF+Cat
7	30’ PDGF+Cat
8	60’ PDGF+Cat

Gel 4B (4)

Lane #	Description
1	No addition
2	10’ PDGF
3	30’ PDGF
4	Cat
5	10’ PDGF+Cat
6	30’ PDGF+Cat

Raw images for Figs.4E

Fig 4E (1)

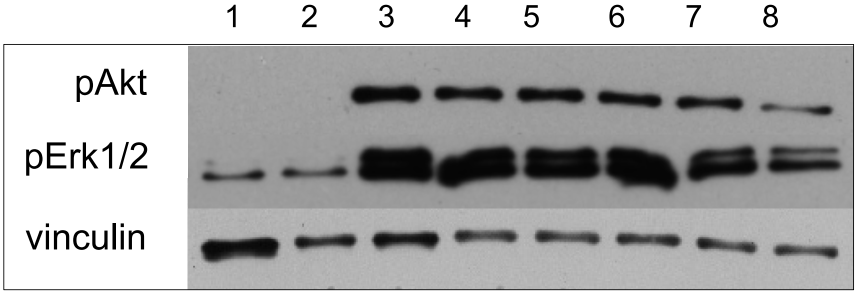

Fig 4E (2)

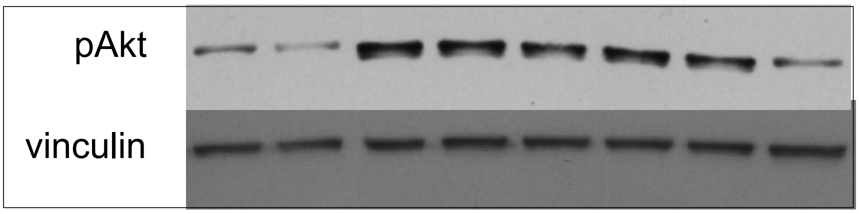

Fig 4E (3)

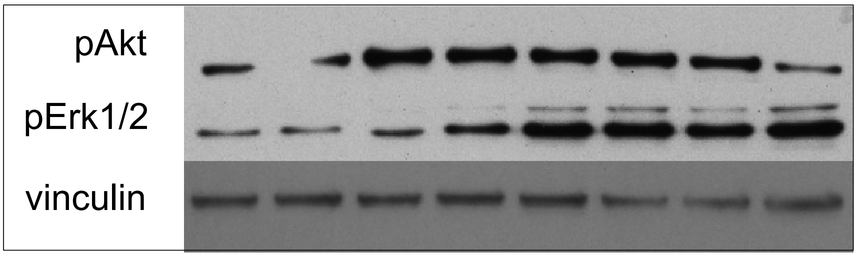

Gel 4E (1)

Lane #	Description
1	No add
2	Apo
3	60’ PDGF
4	60’ PDGF+Apo
5	30’ PDGF
6	30’ PDGF+Apo
7	10’ PDGF
8	10’ PDGF+Apo

Gel 4E (2)

Lane #	Description
1	No add
2	Apo
3	60’ PDGF
4	60’ PDGF+Apo
5	30’ PDGF
6	30’ PDGF+Apo
7	10’ PDGF
8	10’ PDGF+Apo

Gel 4E (3)

Lane #	Description
1	No add
2	Apo
3	60’ PDGF
4	60’ PDGF+Apo
5	30’ PDGF
6	30’ PDGF+Apo
7	10’ PDGF
8	10’ PDGF+Apo

Raw images for Figs.4E (continued), 4F, and 4J

Fig 4E (4) and Fig 4F (1)

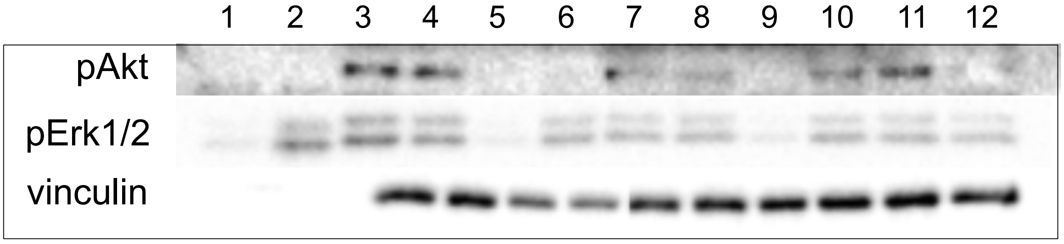

Fig 4F (2)

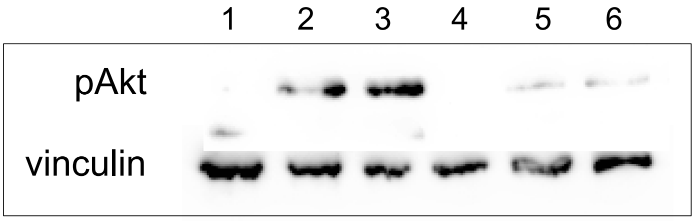

Fig 4F (3) and Fig 4J (1)

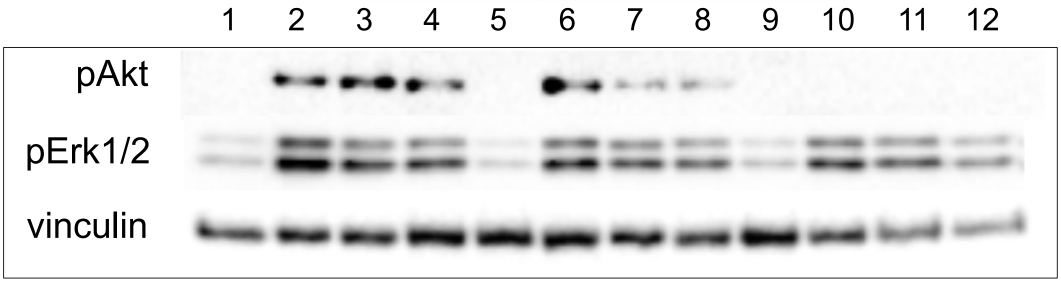

Gel 4E (4) / 4F (1)

Lane #	Description
1	No Add
2	10’ PDGF
3	30’ PDGF
4	60’ PDGF
5	Cat
6	10’ PDGF+Cat
7	30’ PDGF+Cat
8	60’ PDGF+Cat
9	Apo
10	10’ PDGF+Apo
11	30’ PDGF+Apo
12	60’ PDGF+Apo

Gel 4F (2)

Lane #	Description
1	No Add
2	10’ PDGF
3	30’ PDGF
4	Cat
5	10’ PDGF+Cat
6	30’ PDGF+Cat

Gel 4F (3) / 4J (1)

Lane #	Description
1	No Add
2	10’ PDGF
3	30’ PDGF
4	60’ PDGF
5	Cat
6	10’ PDGF+Cat
7	30’ PDGF+Cat
8	60’ PDGF+Cat
9	No Add
10	10’ EGF
11	30’ EGF
12	60’ EGF

Raw images for Fig.4I

Fig 4I (1)

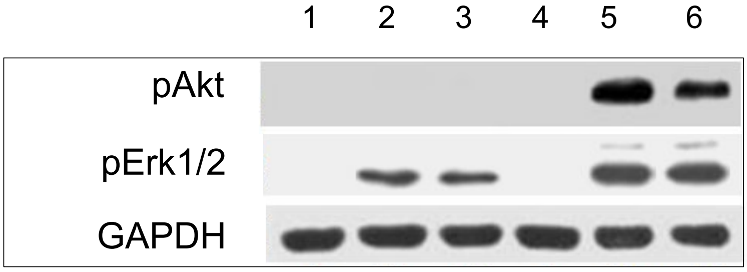

Fig 4I (2)

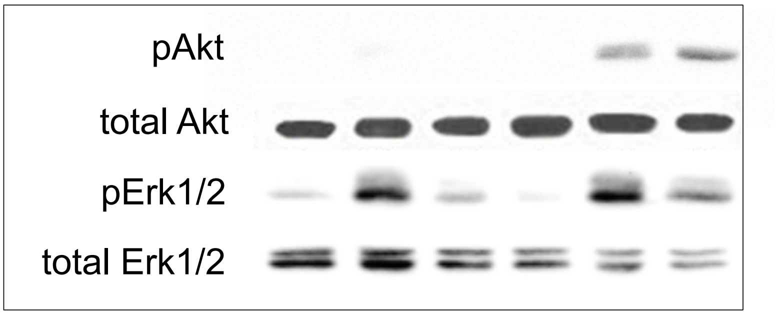

Gel 4I (1)

Lane #	Description
1	No Add
2	10' EGF
3	30' EGF
4	No Add
5	10' PDGF
6	30' PDGF

Gel 4I (2)

Lane #	Description
1	No Add
2	10' EGF
3	30' EGF
4	No Add
5	10' PDGF
6	30' PDGF

Raw images for Fig.5D

Fig 5D (1-3)

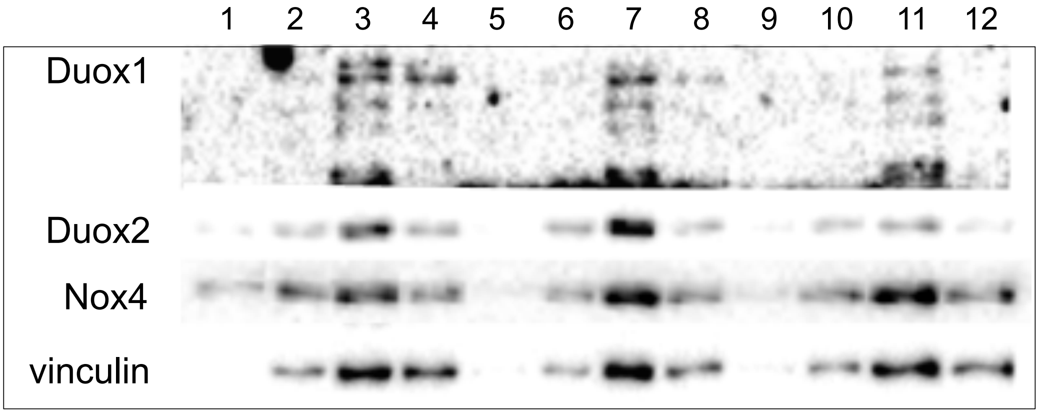

Fig 5D (4-6)

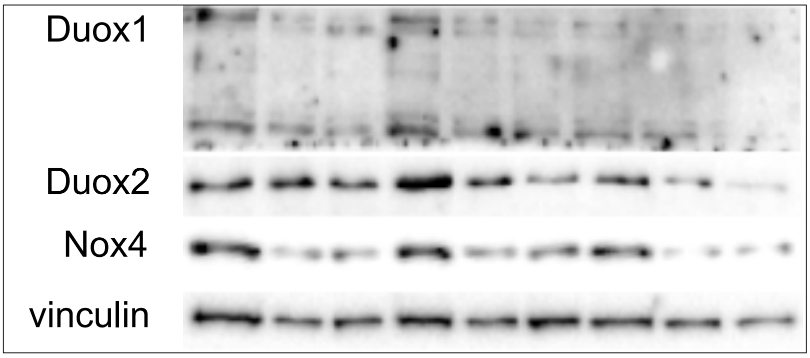

Gel 5D (1-3)

Lane #	Description
1	WT
2	Scramble Set1
3	shNox4 Set1
4	shDuox1 Set1
5	WT
6	Scramble Set2
7	shNox4 Set2
8	shDuox1 Set2
9	WT
10	Scramble Set3
11	shNox4 Set3
12	shDuox1 Set3

Gel 5D (4-6)

Lane #	Description
1	Scramble Set4
2	shNox4 Set4
3	shDuox1 Set4
4	Scramble Set5
5	shNox4 Set5
6	shDuox1 Set5
7	Scramble Set6
8	shNox4 Set6
9	shDuox1 Set6

Raw images for Fig.5F

Fig 5F (1)

Fig 5F (2-3)

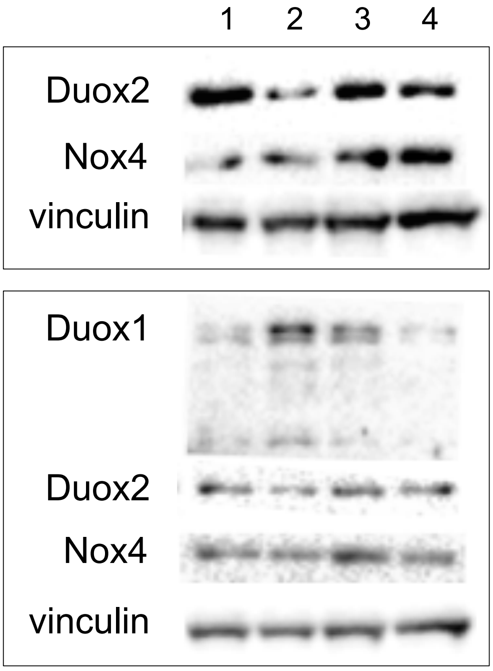

Gel 5F (1)

Lane #	Description
1	NT Set1
2	siDuox2 #1 Set1
3	siDuox1 Set1
4	siDuox2 #2 Set1

Gel 5F (2)

Lane #	Description
1	NT Set2
2	siDuox2 #1 Set2
3	siDuox1 Set2
4	siDuox2 #2 Set2

siDuox1#1 was used in experiments for Fig.6B

Raw images for Fig.5F (continued)

Fig 5F (5-7)

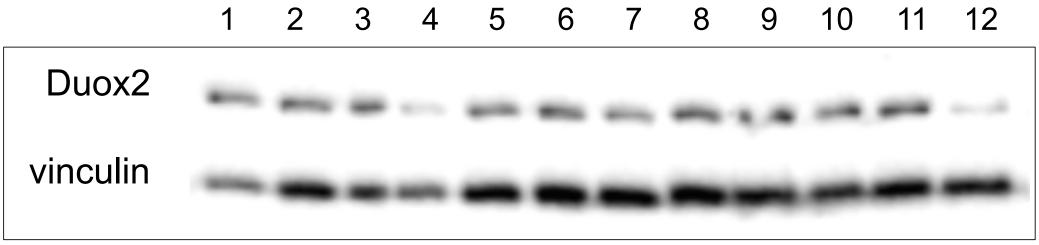

Fig 5F (8)

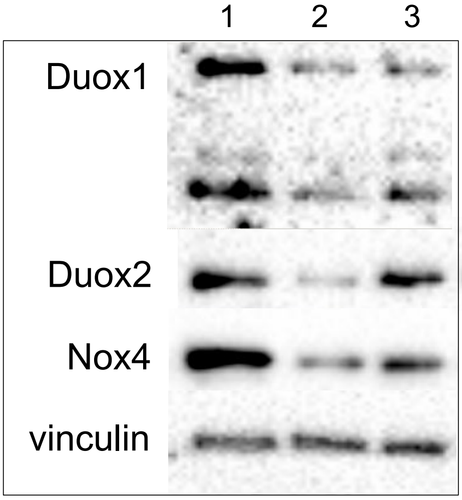

Gel 5F (3-5)

Lane #	Description
1	NT Set5
2	siNox4 Set5
3	siDuox1 Set5
4	siDuox2 #1 Set5
5	NT Set6
6	siNox4 Set6
7	siDuox1 Set6
8	siDuox2#1 Set6
9	NT Set7
10	siNox4 Set7
11	siDuox1 Set7
12	siDuox2#1 Set7

Gel 5F (6)

Lane #	Description
1	NT Set8
2	siDuox2 #1 Set8
3	siDuox1 Set8

Raw images for Fig.5H

Fig 5H (1-2)

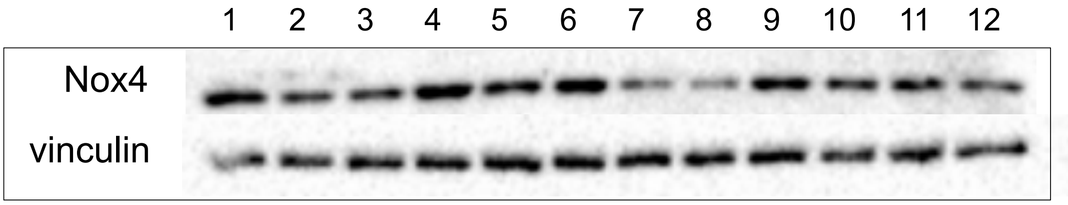

Fig 5H (3-4)

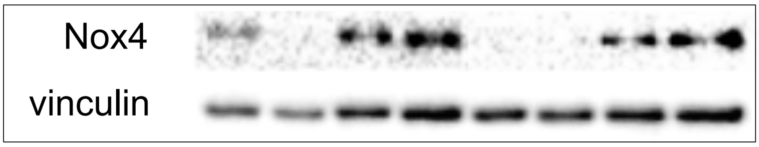

Gel 5H (1-2)

Lane #	Description
1	siNT Set1
2	siNox4#1 Set1
3	siNox4#2 Set1
4	siDuox1#1 Set1
5	siDuox1#2 Set1
6	siDuox2 Set1
7	siNT Set2
8	siNox4#1 Set2
9	siNox4#2 Set2
10	siDuox1#1 Set2
11	siDuox1#2 Set2
12	siDuox2 Set2

Gel 5H (3-4)

Lane #	Description
1	siNT Set1
2	siNox4#1 Set1
3	siDuox1#1 Set1
4	siDuox2 Set1
5	siNT Set2
6	siNox4#1 Set2
7	siDuox1#1 Set2
8	siDuox2 Set2

siNox4#1 and siDuox1#1 were used in experiments for Fig.6C

Raw images for Fig.6A

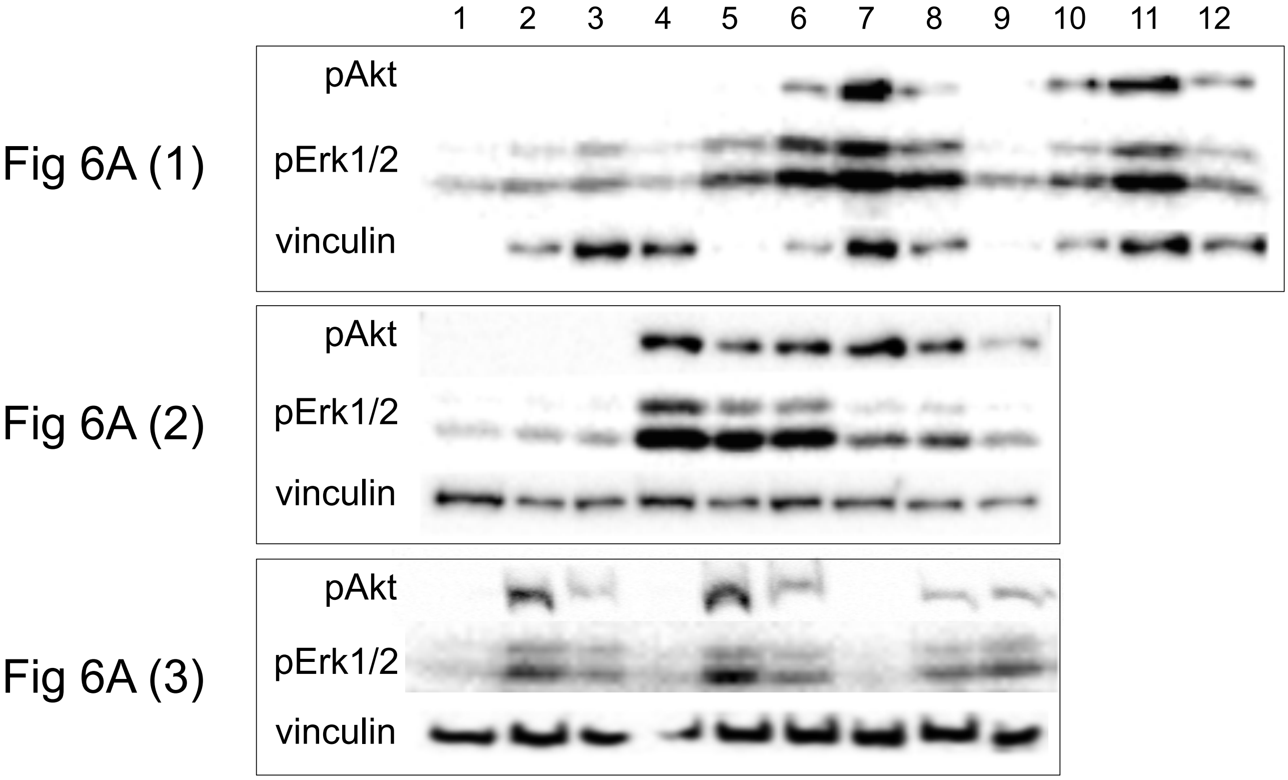

Gel 6A (1)

Lane #	Description
1	WT No addition
2	Scramble No addition
3	shNox4 No addition
4	shDuox1 No addition
5	WT 10’ PDGF
6	Scramble 10’ PDGF
7	shNox4 10’ PDGF
8	shDuox1 10’ PDGF
9	WT 30’ PDGF
10	Scramble 30’ PDGF
11	shNox4 30’ PDGF
12	shDuox1 30’ PDGF

Gel 6A (2)

Lane #	Description
1	Scramble No addition
2	shNox4 No addition
3	shDuox1 No addition
4	Scramble 10’ PDGF
5	shNox4 10’ PDGF
6	shDuox1 10’ PDGF
7	Scramble 30’ PDGF
8	shNox4 30’ PDGF
9	shDuox1 30’ PDGF

Gel 6A (3)

Lane #	Description
1	Scramble No addition
2	Scramble 10’ PDGF
3	Scramble 30’ PDGF
4	shNox4 No addition
5	shNox4 10’ PDGF
6	shNox4 30’ PDGF
7	shDuox1 No addition
8	shDuox1 10’ PDGF
9	shDuox1 30’ PDGF

Raw images for Fig.6B

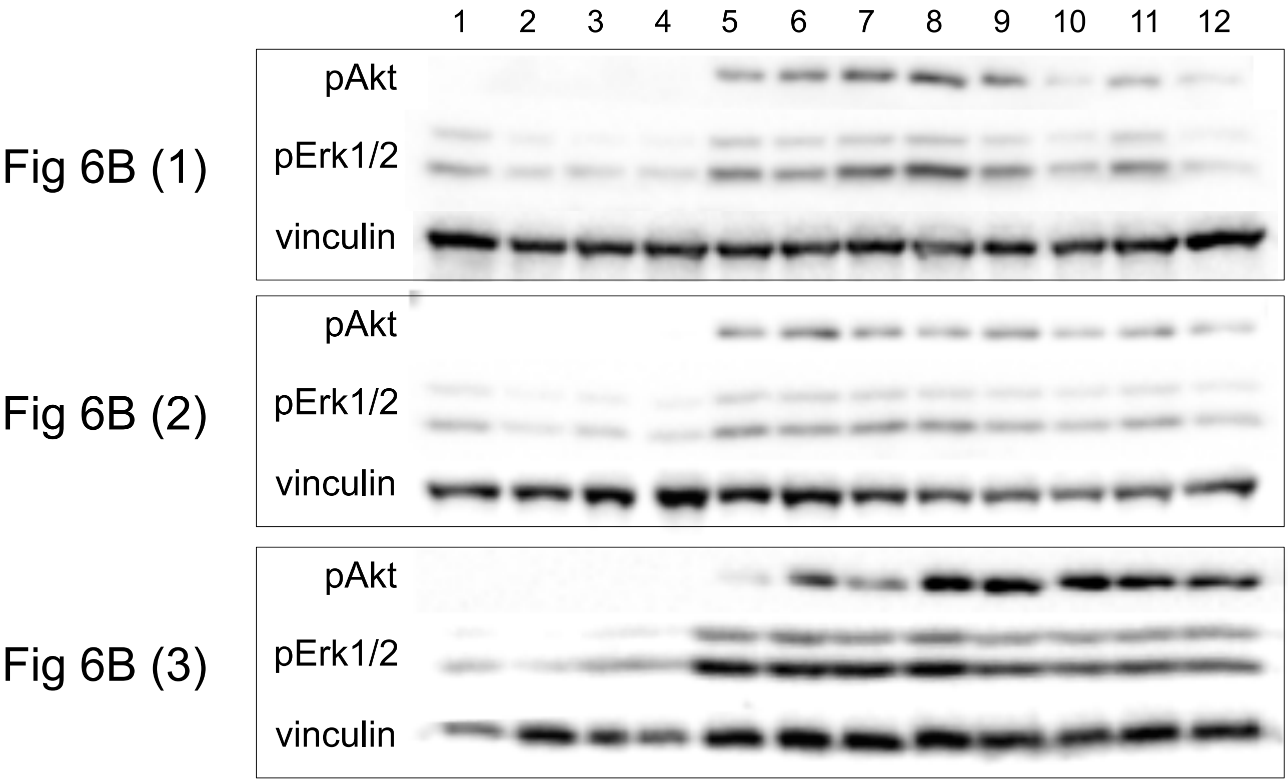

Gel 6B (1)

Lane #	Description
1	siNT No addition
2	siNox4 No addition
3	siDuox1#1 No add
4	siDuox2 No addition
5	siNT 10’ PDGF
6	siNox4 10’ PDGF
7	siDuox1#1 10’ PDGF
8	siDuox2 10’ PDGF
9	siNT 30’ PDGF
10	siNox4 30’ PDGF
11	siDuox1#1 30’ PDGF
12	siDuox2 30’ PDGF

Gel 6B (2)

Lane #	Description
1	siNT No addition
2	siNox4 No addition
3	siDuox1#1 No add
4	siDuox2 No addition
5	siNT 10’ PDGF
6	siNox4 10’ PDGF
7	siDuox1#1 10’ PDGF
8	siDuox2 10’ PDGF
9	siNT 30’ PDGF
10	siNox4 30’ PDGF
11	siDuox1#1 30’ PDGF
12	siDuox2 30’ PDGF

Gel 6B (2)

Lane #	Description
1	siNT No addition
2	siNox4 No addition
3	siDuox1#1 No add
4	siDuox2 No addition
5	siNT 10’ PDGF
6	siNox4 10’ PDGF
7	siDuox1#1 10’ PDGF
8	siDuox2 10’ PDGF
9	siNT 30’ PDGF
10	siNox4 30’ PDGF
11	siDuox1#1 30’ PDGF
12	siDuox2 30’ PDGF

Raw images for Fig.6C

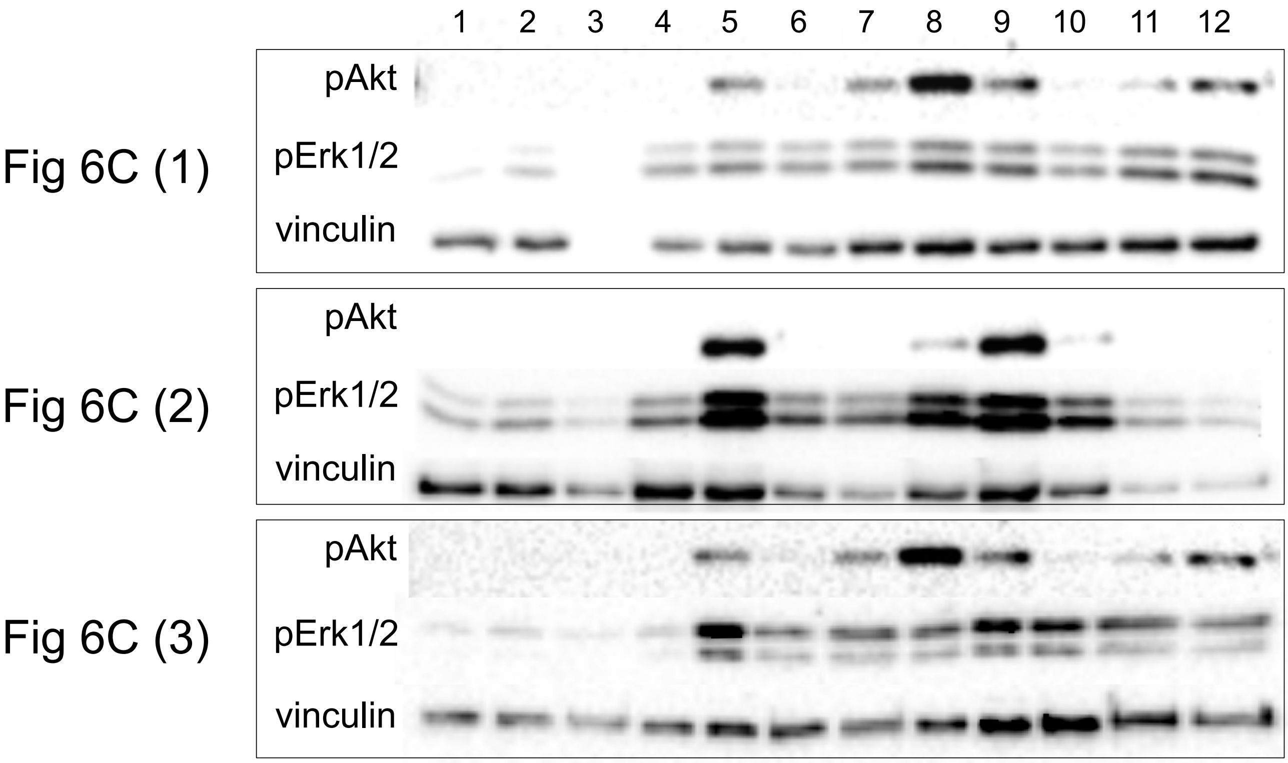

Gel 6C (1)

Lane #	Description
1	siNT No add
2	siNox4#1 No add
3	siDuox1#1 No add
4	siDuox2 No addition
5	siNT 30' PDGF
6	siNox4#1 30' PDGF
7	siDuox1#1 30' PDGF
8	siDuox2 30' PDGF
9	siNT 60' PDGF
10	siNox4#1 60' PDGF
11	siDuox1#1 60' PDGF
12	siDuox2 60' PDGF

Gel 6C (2)

Lane #	Description
1	siNT No add
2	siNox4#1 No add
3	siDuox1#1 No add
4	siDuox2 No addition
5	siNT 30' PDGF
6	siNox4#1 30' PDGF
7	siDuox1#1 30' PDGF
8	siDuox2 30' PDGF
9	siNT 60' PDGF
10	siNox4#1 60' PDGF
11	siDuox1#1 60' PDGF
12	siDuox2 60' PDGF

Gel 6C (2)

Lane #	Description
1	siNT No add
2	siNox4#1 No add
3	siDuox1#1 No add
4	siDuox2 No addition
5	siNT 30' PDGF
6	siNox4#1 30' PDGF
7	siDuox1#1 30' PDGF
8	siDuox2 30' PDGF
9	siNT 60' PDGF
10	siNox4#1 60' PDGF
11	siDuox1#1 60' PDGF
12	siDuox2 60' PDGF
